# Optimization of Cavity-Based Negative Images to Boost Docking Enrichment in Virtual Screening

**DOI:** 10.1021/acs.jcim.1c01145

**Published:** 2022-02-08

**Authors:** Sami T. Kurkinen, Jukka V. Lehtonen, Olli T. Pentikäinen, Pekka A. Postila

**Affiliations:** † Institute of Biomedicine, Integrative Physiology and Pharmacy, University of Turku, FI-20014 Turku, Finland; ‡ Aurlide Ltd., FI-21420 Lieto, Finland; § InFLAMES Research Flagship Center, University of Turku, FI-20014 Turku, Finland; ∥ Structural Bioinformatics Laboratory, Biochemistry, Faculty of Science and Engineering, Åbo Akademi University, FI-20500 Turku, Finland; ⊥ InFLAMES Research Flagship Center, Åbo Akademi University, FI-20500 Turku, Finland

## Abstract

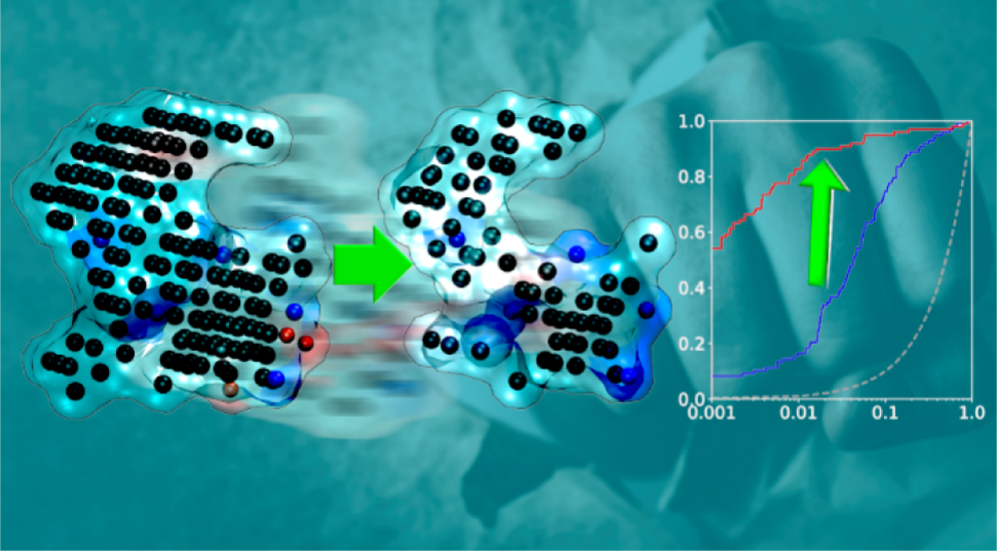

Molecular docking is a key in silico method used routinely in modern drug discovery projects. Although docking provides high-quality ligand binding predictions, it regularly fails to separate the active compounds from the inactive ones. In negative image-based rescoring (R-NiB), the shape/electrostatic potential (ESP) of docking poses is compared to the negative image of the protein’s ligand binding cavity. While R-NiB often improves the docking yield considerably, the cavity-based models do not reach their full potential without expert editing. Accordingly, a greedy search-driven methodology, brute force negative image-based optimization (BR-NiB), is presented for optimizing the models via iterative editing and benchmarking. Thorough and unbiased training, testing and stringent validation with a multitude of drug targets, and alternative docking software show that BR-NiB ensures excellent docking efficacy. BR-NiB can be considered as a new type of shape-focused pharmacophore modeling, where the optimized models contain only the most vital cavity information needed for effectively filtering docked actives from the inactive or decoy compounds. Finally, the BR-NiB code for performing the automated optimization is provided free-of-charge under MIT license via GitHub (https://github.com/jvlehtonen/brutenib) for boosting the success rates of docking-based virtual screening campaigns.

## Introduction

Despite the pivotal role of molecular docking in protein structure-based drug discovery,^1−4^ the docking-based screening often falls short of expectations. The problem is not necessarily the inadequacies of docking sampling; instead, the default scoring cannot rank the bioactive binding poses at the top and, thus, effectively identify the active ligands. Several rescoring, consensus scoring, or force field-based post-processing methodologies aim to fix the problem, but, so far, their successes have been case specific or costly.^5−9^

**Figure 1:**
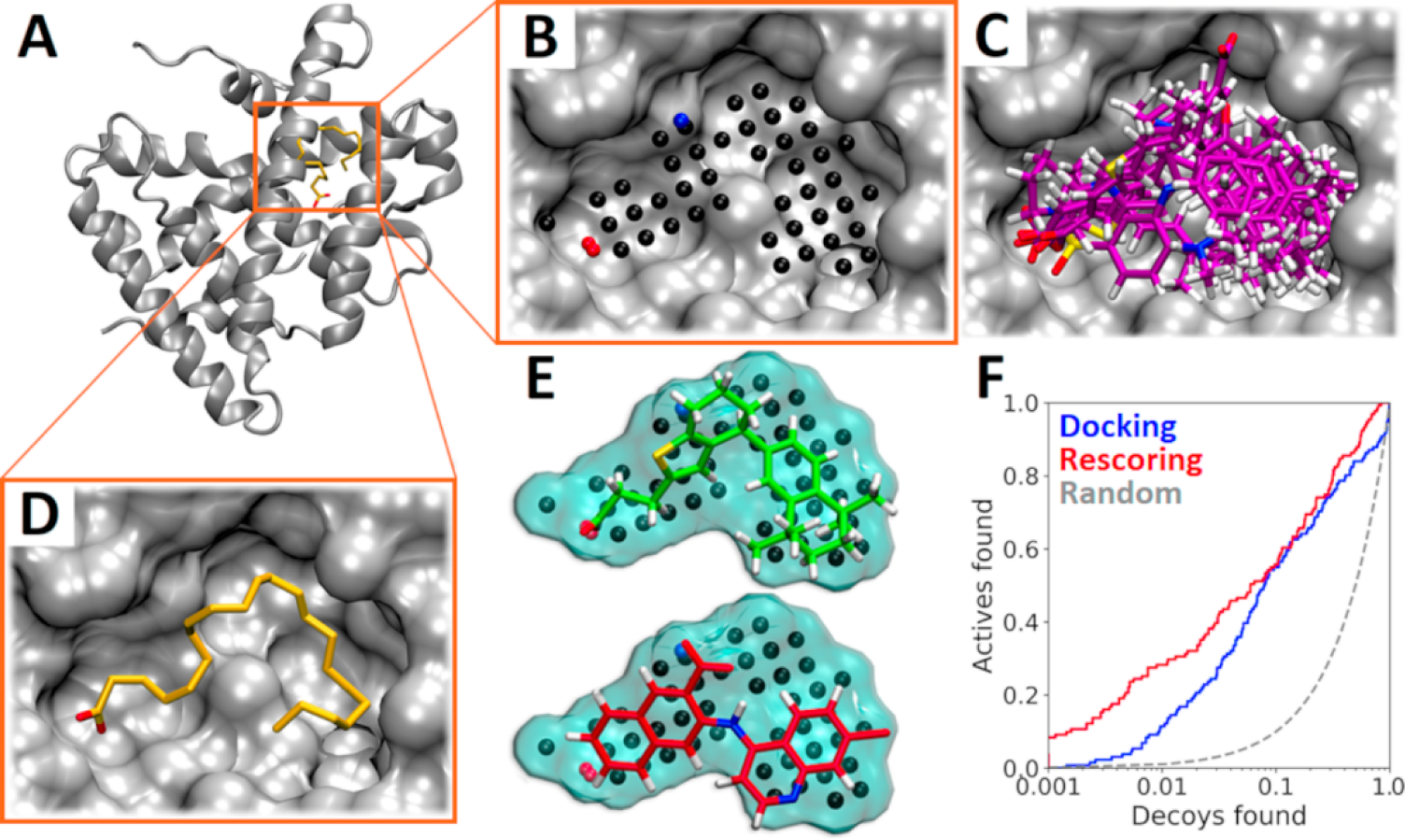
Negative image-based rescoring. (A) Ligand binding cavity of RXRα (gray cartoon; PDB: 1MV9(23)) with a co-crystallized agonist (yellow stick model). (B) NIB model against the cavity cross-section (gray surface). The NIB model, depicting the key cavity shape/electrostatic potential features, is composed of negative (O; red), positive (N; blue), and neutral (C; black) cavity atoms (spheres). (C) Overlay of docking poses are shown for seven active ligands (magenta stick models), where (D) co-crystallized agonist is bound. (E) Best (green) and worst (red) shape/electrostatic matches for two docked actives (stick models) are shown with the NIB model (cyan surface). (F) Receiver operating characteristic (ROC) curves show that the R-NiB (red line) boosts the docking yield (blue line).

A potential solution to this persistent problem is to score accurately the shape complementarity between the docked ligand and its target protein’s binding cavity.^10−12^ Negative image-based rescoring (R-NiB; Figure 1) is a cavity-based docking rescoring methodology that takes on this challenge by focusing squarely on the shape/electrostatic potential (ESP) complementarity.^13^ R-NiB has been shown to improve the yields with several docking algorithms (e.g., DOCK,^14^ GLIDE,^15,16^ or PLANTS^17^) and multiple drug targets, such as neuraminidase (NEU) and retinoid X receptor alpha (RXRα; Figure 1A).^18^

**Figure 2:**
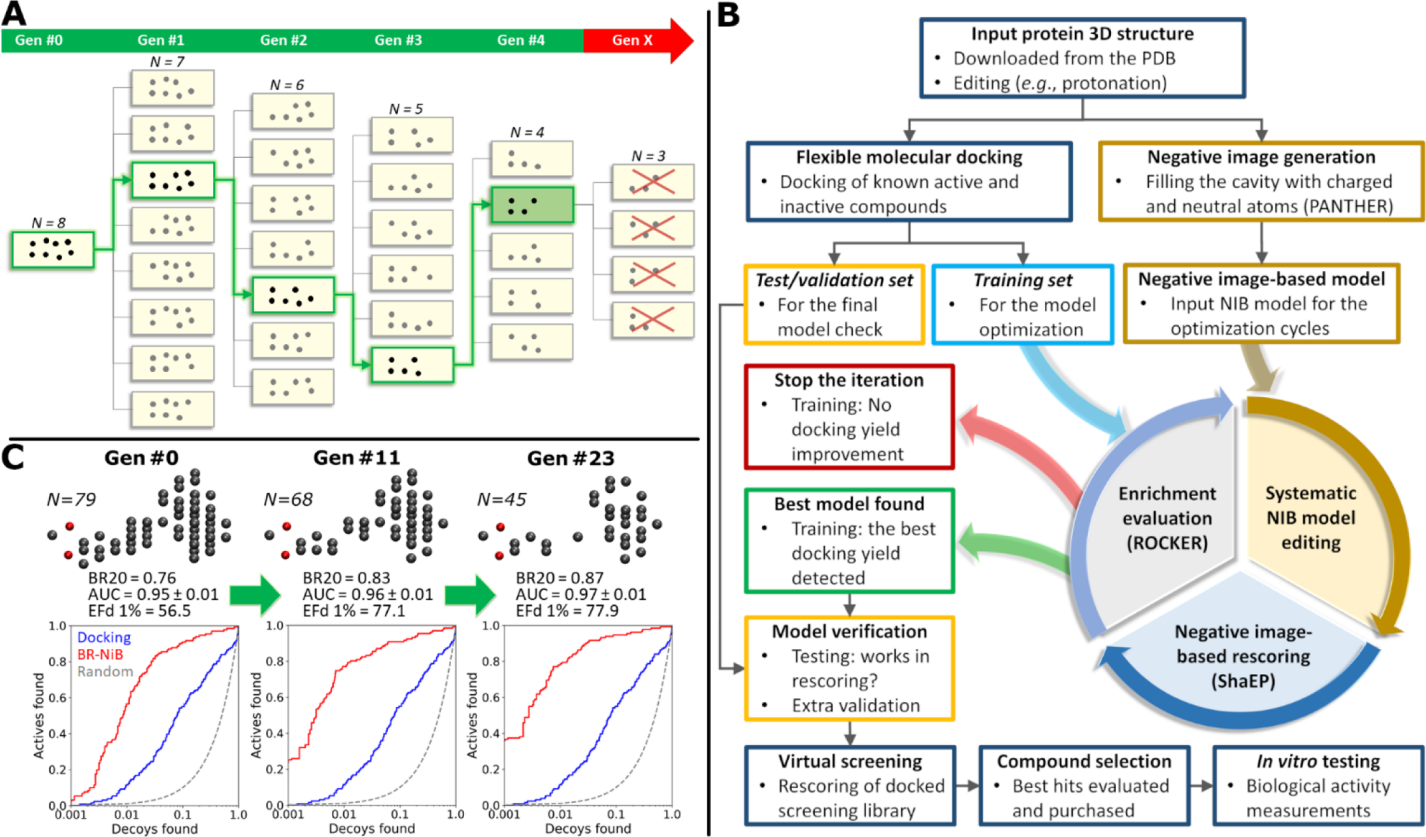
Brute force negative image-based optimization. (A) During the first generation (Gen #1) of the optimization, each cavity atom is removed from the initial NIB model (Gen #0) one at the time to generate eight new seven-atom variants. If one of the variants improves enrichment (node boxed green) more than the other variants or the input in docking rescoring, it is used as the input for another round of editing/benchmarking. Here, the iteration goes through Gens #2–4 as the enrichment improves, and none of the last variants (Gen #5 = Gen #X) improves on the best model (Gen #4; node with a green background). (B) Atom composition evolution is shown for retinoid X receptor alpha for the input (Gen #0), mid-point (Gen #11), and fully optimized (Gen #23) NIB models. The rescoring with the final model (red line) is superior to docking (blue line), as seen in the ROC curves (*x* axis logarithmic). (C) Optimization protocol includes testing and potentially even validation prior to the virtual screening usage.

R-NiB can be performed using free/academic software from start to finish following a straightforward workflow. First, the binding poses of ligands are sampled flexibly against the target’s cavity using a docking algorithm (Figure 1C,D). Second, a negative image-based (NIB) model (Figure 1B) is generated in a mirror image of the cavity using PANTHER^19^ -cavity detection/filling software that was developed for cavity-based rigid docking or NIB screening.^20^ Third, the shape/ESP of the NIB model, containing partially negative, positive, and neutral cavity atoms (Figure 1D), is compared against the docking poses using the similarity comparison algorithm SHAEP^21^ (Figure 1E). R-NiB typically elicits a decent or excellent docking performance (Figure 1F),^22^ but the model fitness can be improved massively by adjusting the settings or via manual editing.^13,18,22^ Usually, the PANTHER-generated NIB models contain at least a few “extra” cavity atoms that are not providing optimal shape/ESP for the filtering of active ligands from the “inactive” decoys and, thus, the models can be improved by removing some of them. Regardless, it can be perplexing why the removal of a few atoms makes or breaks the rescoring. The aim of this study was to make the model optimization both thorough and automatic following the initial docking and NIB model generation (Figure 2; Videos S1 and S2).

Machine learning techniques are emerging for both compound-centric and structure-based computer-aided drug discovery.^24−27^ However, due to the ultra-fast speed of R-NiB (∼2–4 ms/cmpd.),^13^ the optimization could be performed via a greedy search-driven method labeled here as brute force negative image-based optimization (BR-NiB; Figure 2).^28^ In BR-NiB, the effect of each cavity atom on the NIB model (Figure 1B) is evaluated systematically by iterative removal and rescoring (Figure 2; Videos S1 and S2). In pruning, the atoms are removed one at a time and the fitness of each new (-1 atom) model variant is tested with SHAEP^21^ using the docked training set. In branching, the model producing the best enrichment goes into the next round of iterative removal and rescoring (Figure 2). Although not all permutations are tested in this heuristic scheme, the greedy search is repeated for as many generations as the yield improves (Figure 2).

BR-NiB (Figure 2) was tested thoroughly with seven Database of Useful (Docking) Decoys-Enhanced (DUD-E) database^29^ sets for finding the best optimization practices (Table S1 in the Supporting Information). Likewise, seven equivalent DUD^30^ sets were tested. To increase the target diversity and vary database design, extra testing was done with 10 Maximum Unbiased Validation (MUV^31^) sets, 5 more DUD-E sets, 5 DUDE-Z sets, and 5 Extrema sets.^32^ In docking rescoring or testing, the optimized NIB models improved the overall enrichment consistently and substantially; importantly, the early enrichment was typically massively boosted. Although BR-NiB is scalable to supercomputers, in most cases, it can be performed with moderately sized training sets using a desktop computer. Moreover, the effectiveness of BR-NiB is not docking algorithm-specific, and the optimized models can even be cross-used effectively. Finally, the optimized models for two DUD-E targets were validated with newly crafted validation sets, in which minute amounts of active ligands at varying potency levels were mixed into an enormous drug-like small-molecule library. The success in this validation step, having extremely low odds of finding hits by chance, demonstrates that BR-NiB has real potential in helping actual drug discovery projects.

In summary, if a reliable training set and a high-quality target protein 3D structure are available, the BR-NiB-optimized models present a tangible way for improving the effectiveness of docking-based virtual screening campaigns via ultra-fast rescoring.

## Results

### Using Greedy Search to Boost Negative Image-Based Rescoring

Negative image-based rescoring (Figure 1) produces excellent or at least moderate enrichment for docking (Gen #0 in Tables S2–S3).^13,18^ Although the NIB models can be tested prior to their docking screening use, as it stands, the R-NiB protocol does not include optimization steps assuring consistently excellent yields.^18,22^ The goal was to develop fast and easy-to-use method for optimizing the models by utilizing the categorical active/inactive ligand sets in a systematic and yet cost-effective manner. In BR-NiB (Figure 2; Videos S1 and S2), the effect of each cavity atom is estimated by removing them systematically from the model and then benchmarking each new (-1 cavity atom) model variant. By automating the optimization, BR-NiB takes full advantage of the ultra-fast SHAEP rescoring and the existing compound activity data.

### Selecting the Target Metric for the Optimization

BR-NiB promotes the removal of those cavity atoms that improve the selected target enrichment metric at any given iteration until the improvement halts (Gen #X in Figure 2; Video S1). Three target metrics, including area under the curve (AUC), early enrichment factor 1% (EFd 1%), and Boltzmann-enhanced discrimination of the receiver operating characteristic (BEDROC)^33^ with alpha value 20 (BR20) were tested using four test sets (Tables S1 and S4), including compounds that are in either active or “inactive” decoy category. Each metric worked better or worse on certain targets than with others. The EFd 1% is the most ill-suited metric for guiding BR-NiB because it is sensitive to changes at the very top of the ranking list and to the size of the training set. As target metrics, AUC or BR20 produce a better overall enrichment than EFd 1% because they allow the models to undergo larger transformations during the greedy search without getting hung up on losing a few actives from the top. Because BR20 produced the best overall results on early enrichment (Table S4), it was chosen as the default target metric for the testing. When plotted against the BR-NiB generations, BR20 acquired smooth gradual improvement; meanwhile, the upward curves of the non-target metrics are more jagged (Figures S2–S3).

### Model Optimization Ensures Consistent Docking Performance Improvement

The proof-of-concept testing of BR-NiB was performed using seven established DUD-E test sets including cyclooxygenase-2 (COX2), retinoid X receptor alpha (RXRα), mineralocorticoid receptor (MR), NEU, phosphodiesterase 5 (PDE5), estrogen receptor (ER), and peroxisome proliferator-activated receptor gamma (PPARγ) (Table S1). First, the input NIB models were trained using the complete compound sets using BR20 as the target metric. This naïve approach (100:100 training/test set), lacking random training/test set division, served as a trial run for BR-NiB. Likewise, the naïve testing was repeated for the equivalent but considerably smaller DUD database sets (Table S5).^30^ Second, to avoid bias, the active and “inactive” decoy ligands were divided randomly into training and test sets (Table S1, Figure 2). Two ratios were applied: (1) the 70:30 ratio of training/test sets represents a situation in which there exists a wealth of data for the model training and (2) the 10:90 ratio represents a situation in which the available set is considerably smaller (e.g., nine actives with MR).

BR-NiB treatment improved on docking or R-NiB rescoring yields consistently with both training (Table S6) and test sets (Table 1; Figures S3–S9). More importantly, the improvement of AUC and EFd values indicate that the model fitness could be enhanced both regarding the overall and early enrichment. At its best, the EFd 1% improvement with 100:100 and 70:30 training/test set division surpassed the original docking over 20-fold (e.g., 100:100 NEU set with the 50/50 shape/ESP weight; 70:30 MR set with the only shape score). The EFd 1% improvement of BR-NiB over docking varied between 1.3- and 25.3-fold (excluding PPARγ), while the BR20 values acquired only 1.2- to 3.1-fold increase. The optimization worked best when relying only on the shape similarity for COX2, PDE5, and PPARγ (black lines in Figures S8–S9; Table 1).

**Table 1:** Docking and Brute Force Negative Image-Based Rescoring with the Test Sets^a^

train/test^b^	method^c^	yield	COX2	RXRα	MR	NEU	PDE5	ER	PPARγ
100:100	docking	AUC	0.66 ± 0.01	0.77 ± 0.02	0.55 ± 0.03	0.85 ± 0.02	0.78 ± 0.01	0.74 ± 0.01	0.85 ± 0.01
		EFd 1%	5.7	11.5	3.2	4.1	11.3	21.7	24.2
		EFd 5%	21.6	37.4	19.1	32.7	28.1	36.6	57.0
		BR20	0.22	0.35	0.17	0.29	0.28	0.36	0.49
	BR-NiB	AUC	***0.78* ± *0.01***	***0.97 ± 0.01***	***0.72 ± 0.03***	***0.97 ± 0.01***	***0.82* ± *0.01***	***0.81 ± 0.01***	0.83 ± 0.01
		EFd 1%	***32.2***	***77.9***	***33.0***	***82.7***	***20.1***	***40.9***	***25.8***
		EFd 5%	***52.2***	***91.6***	***48.9***	***91.8***	***41.0***	***54.6***	52.7
		BR20	***0.50***	***0.87***	***0.49***	***0.89***	***0.39***	***0.55***	0.47
	BR-NiB + shape only	AUC	***0.83 ± 0.01***	***0.86* ± *0.02***	***0.73 ± 0.03***	***0.96 ± 0.01***	***0.87 ± 0.01***	0.68 ± 0.02	0.85 ± 0.01
		EFd 1%	***38.5***	***48.1***	***30.9***	***77.6***	***27.6***	***33.4***	***38.4***
		EFd 5%	***57.5***	***71.8***	***40.4***	***87.8***	***50.8***	***43.9***	***58.3***
		BR20	***0.57***	***0.66***	***0.42***	***0.85***	***0.46***	***0.44***	***0.57***
70:30	docking	AUC	0.66 ± 0.02	0.77 ± 0.03	0.53 ± 0.04	0.85 ± 0.03	0.77 ± 0.02	0.77 ± 0.02	0.85 ± 0.01
		EFd 1%	5.5	12.1	1.5	2.9	10.8	28.2	21.2
		EFd 5%	19.5	37.4	15.4	32.4	27.0	34.8	61.0
		BR20	0.20	0.35	0.14	0.29	0.27	0.42	0.50
	BR-NiB	AUC	***0.79 ± 0.02***	***0.98 ± 0.02***	***0.77 ± 0.05***	***0.93 ± 0.03***	***0.82* ± *0.02***	***0.82 ± 0.02***	0.81 ± 0.02
		EFd 1%	***35.9***	***82.5***	***31.0***	***56.7***	***15.0***	***38.2***	***28.8***
		EFd 5%	***49.2***	***92.5***	***48.3***	***76.7***	***40.0***	***56.4***	49.3
		BR20	***0.50***	***0.86***	***0.47***	***0.73***	***0.35***	***0.53***	0.46
	BR-NiB + shape only	AUC	***0.82 ± 0.02***	***0.86* ± *0.04***	***0.74 ± 0.05***	***0.93 ± 0.03***	***0.87 ± 0.02***	0.69 ± 0.03	0.83 ± 0.02
		EFd 1%	***36.7***	***50.0***	***37.9***	***66.7***	***18.3***	28.2	***38.4***
		EFd 5%	***56.3***	***72.5***	***44.8***	***83.3***	***41.7***	***47.3***	54.1
		BR20	***0.54***	***0.69***	***0.49***	***0.79***	***0.41***	***0.46***	***0.54***
10:90	docking	AUC	0.67 ± 0.02	0.77 ± 0.03	0.56 ± 0.03	0.85 ± 0.03	0.77 ± 0.01	0.73 ± 0.02	0.85 ± 0.01
		EFd 1%	5.6	13.6	3.5	3.4	10.9	22.6	23.9
		EFd 5%	22.2	39.8	17.6	31.5	28.4	36.8	56.4
		BR20	0.21	0.37	0.17	0.28	0.28	0.36	0.49
	BR-NiB	AUC	***0.77* ± *0.01***	***0.95 ± 0.01***	***0.80 ± 0.03***	***0.93 ± 0.02***	0.75 ± 0.01	0.66 ± 0.02	0.81 ± 0.01
		EFd 1%	***30.2***	***72.0***	***11.8***	***56.2***	5.3	***33.9***	11.9
		EFd 5%	***50.5***	***84.7***	***38.8***	***75.3***	20.1	***44.6***	42.4
		BR20	***0.48***	***0.80***	***0.34***	***0.71***	0.20	***0.45***	0.37
	BR-NiB + shape only	AUC	***0.82 ± 0.01***	0.68 ± 0.03	***0.73* ± *0.03***	***0.92 ± 0.02***	***0.79 ± 0.01***	0.75 ± 0.02	0.81 ± 0.01
		EFd 1%	***39.6***	***22.0***	***16.5***	***49.4***	***14.8***	***30.7***	***24.1***
		EFd 5%	***57.5***	34.7	***35.3***	***69.7***	***37.0***	***45.2***	42.0
		BR20	***0.55***	0.36	***0.34***	***0.66***	***0.33***	***0.46***	0.42

a The best values are underlined. The rescoring values are shown in bold and italics, if improved in comparison to the docking of each set (70, 30, 10, or 90%). Only those AUC values that are outside the error margin are highlighted. The Wilcoxon statistic^34^ was used for the AUC error estimation.

b Training/test set ratios (100:100, 70:30, and 10:90): the percentage of ligands used in the training (100, 70, and 10%) in relation to the percentage used in the testing (100, 30, and 90%).

c Methods: flexible docking (PLANTS) and BR-NiB either with the equal shape/ESP (0.5/0.5) weight or the shape only (1.0/0.0).

BR-NiB improved on the docking yield also for the 10:90 sets regarding both training (Table S6) and testing (Table 1). The EFd 1% improvement varied between 1.4- and 16.5-fold and BR20 between 1.1- and 3.5-fold (Table 1; Figures S8–S9). In comparison to straight-up R-NiB (Gen #0 in Tables S2–S3), BR-NiB provided 1.2-fold to 7.6-fold (10:90 MR and 100:100 NEU sets with the only shape, respectively) and 1.1- to 2.0-fold (e.g., 70:30 RXRα and 100:100 MR sets with the 50/50 shape/ESP weight) improvement in EFd 1%. On average, BR-NiB improved the AUC values of docking from 0.74 to 0.83 (100:100 and 70:30 sets) and 0.74 to 0.81 (10:90 sets). With PPARγ, the docking results were not improved using the 50/50 weight of shape/ESP scoring; however, the EFd 1% value was improved 1.5-fold with both 100:100 and 70:30 ratios using the only shape similarity scoring. In addition, with PDE5, the results for the 10:90 ratio was improved only, when excluding ESP similarity from the optimization. Interestingly, the docking did even worse with an alternative PPARγ structure with 70:30 sets, whereas the opposite was true for BR-NiB regardless of the applied shape/ESP weight (Table 2).

**Table 2:** Extra Test Set Results on Five DUD-E Targets and the Alternative PPARγ Structure^a^

train/test^b^	method^c^	yield	AKT1	DRD3	ACES	COMT	FAK1	PPARγ^d^
**70:30**	docking	AUC	0.70 ± 0.03	0.60 ± 0.03	0.39 ± 0.02	0.68 ± 0.08	0.80 ± 0.05	***0.83 ± 0.02***
		EFd 1%	5.7	0.7	0.0	7.7	20.0	18.4
		EFd 5%	22.7	6.3	2.9	23.1	36.7	46.9
		BR20	0.25	0.06	0.03	0.22	0.38	0.42
	BR-NiB	AUC	***0.81 ± 0.03***	***0.79 ± 0.02***	***0.69 ± 0.03***	0.67 ± 0.08	***0.95 ± 0.03***	**0.86 ± 0.02** (0.85 ± 0.02)
		EFd 1%	***20.5***	***25.7***	***18.4***	***15.4***	***73.3***	***43.8*** (38.4)
		EFd 5%	***53.4***	***47.9***	***33.1***	***30.8***	***83.3***	***61.0*** (58.9)
		BR20	***0.46***	***0.45***	***0.32***	***0.30***	***0.83***	***0.59*** (0.57)

a The best values are shown in bold and italics for the test sets (30%). Only those AUC values that are outside the error margin are highlighted. The Wilcoxon statistic^32^ was used for the AUC error estimation. An alternative protein 3D structure (PDB: 2GTK) was used for PPARγ docking and BR-NiB optimization.

b Training/test set ratio (70:30): the percentage of ligands used in the training (70%) in relation to the percentage used in the testing (30%).

c Methods: flexible docking (PLANTS) and BR-NiB with the equal shape/ESP (0.5/0.5) weight.

d The shape only (1.0/0.0 of shape/ESP) weight of scoring results is shown in parentheses for PPARγ.

Limited compound sets can be a problem for validating the BR-NiB performance. For example, the yield improvements with the DUD sets were excellent but due to relatively low compound numbers training/test set divisions were omitted (Table S5). Moreover, when the training set is tiny, the optimized model is likely to become too specialized or even overfitted to match closely only those ligands that are present. Nevertheless, the BR-NiB execution is clearly worthwhile even with limited training sets. In fact, a large training set does not automatically guarantee the best results. As good or even better enrichment can sometimes be achieved with the smaller sets (70:100 and 10:100 in Table S8) in comparison to the complete training sets (100:100 in Table 1).

### Merging Models for the Optimization

**Figure 3:**
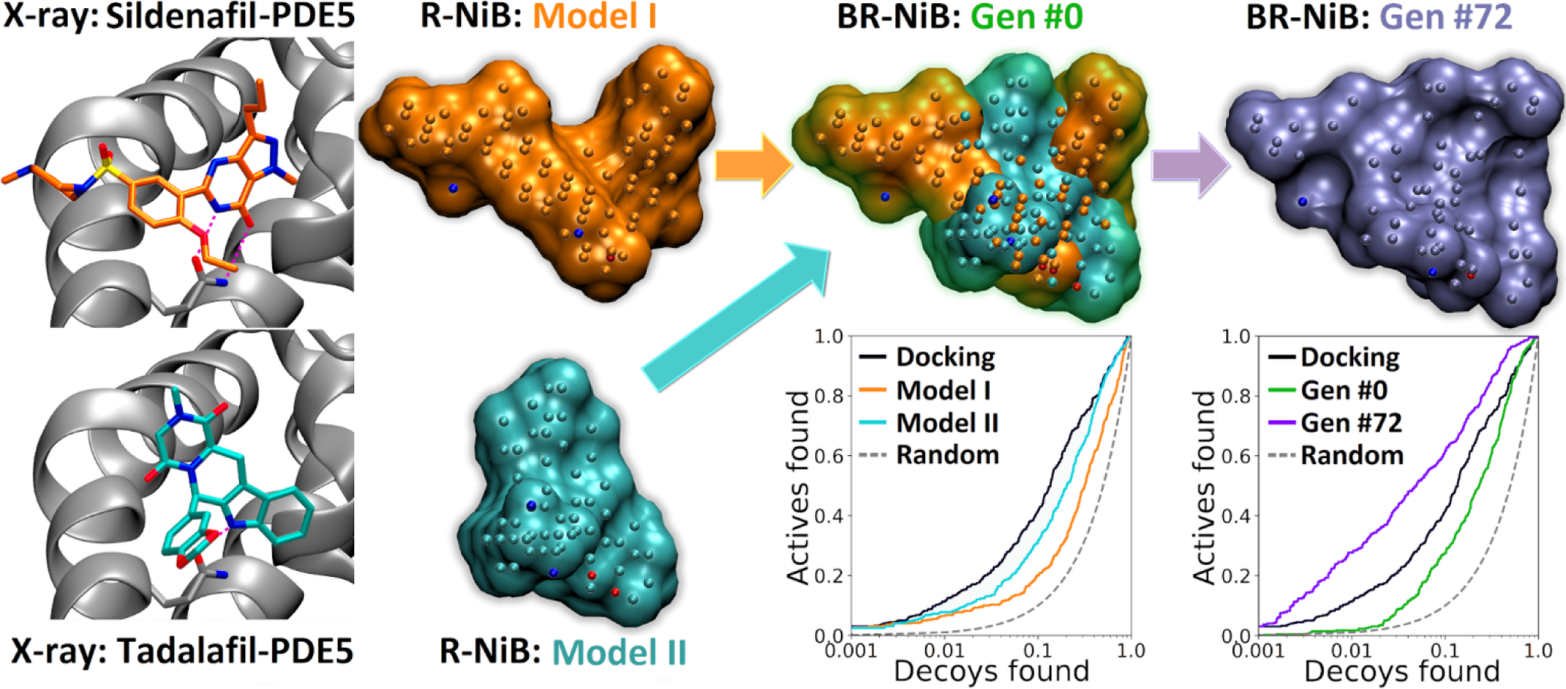
Fusing negative image-based models to boost BR-NiB for PDE5. If R-NiB (Figure 1) was done using an NIB model focusing on sildenafil- (Model I; orange line; PDB: 1UDT(35)) or tadalafil-specific (Model II; cyan line; PDB: 1XOZ(36)) binding volume, the PLANTS scoring (black line) worked better in comparison. In fact, ROC curves indicate that the R-NiB treatment worsened the yield. The fusion of these models lowered the enrichment even further (green line; Gen #0); however, the BR-NiB (magenta line; Gen #72) of the hybrid model boosted the docking performance substantially. See Figure 1 for interpretation.

With targets such as PDE5 that have spacious cavities, it is difficult to generate in one go, a single NIB model that recognizes all active ligand sub-groups. If the PDE5 model is limited to sildenafil- (Model I in Figure 3) or tadalafil-bound (Model II in Figure 3) cavity volume, the docking performance was not typically improved (Table S3). The BR-NiB processing that relied solely on the shape similarity provided a limited boost to the EFd values on these individual PDE5 models. While the fusion of two models did not improve the R-NiB results, the optimization of this combined model worked on every level (Table 1 and Table S3; Figure 3). For example, with a 70:30 ratio, the EFd 1% improved 1.4- and 1.7-fold with the 50/50 weight of shape/ESP and shape only similarity score, respectively, over docking. Similarly, BR-NiB improved the AUC values of PDE5 docking from 0.77 ± 0.02 to 0.82 ± 0.02 (50/50 shape/ESP) and 0.87 ± 0.02 (only shape). In other words, docking performance can be improved even in difficult cases by fusing multiple models together for the optimization.

### Changes to the Model Composition and Fitness during Optimization

BR-NiB improves the fitness gradually atom-by-atom until the best model is found (Figures S2 and S3). With RXRα, the input model composed of 79 cavity atoms and the best enrichment was acquired at Gen #23 with a model composed of 45 atoms (Figure 2). The model at the Gen #0 produced already substantial improvement over docking (e.g., AUC: 0.77 ± 0.02 vs 0.95 ± 0.01; Tables S2 vs 1); however, the optimization pushed the yield gains much higher (Table 1; Figure 2). Whereas R-NiB could improve the EFd 1% of docking from 11.5 to 56.5, the BR-NiB-optimized model generated EFd 1% value of 77.9. The impressive 6.7-fold EFd 1% improvement by BR-NiB over docking is clearly visible, when comparing the ROC curve of docking to the Gen #23 of BR-NiB (Figure 2). Notably, a close-to-optimal NIB model is generated already at the midpoint of the optimization (Gen #11 in Figure 2).

A closer look into the BR-NiB-optimized models, which perform far better in rescoring than the extracted co-crystal ligands (Figure S11), shows that they contain information-related wide spectrum of molecules. The optimization lowers the nonpolar atom content 2–5%-points even when relying solely on the shape similarity, in other words, the mix of dissimilar atomic radii of polar (N/O) and nonpolar atoms (C) assist in depicting the volume optimally (Table S10). While there are target-specific differences, the polar atoms of the optimized models continue to overlap with those chemical groups of ligands that form key bonding interactions (Figure S10B,C).^19^ The surface atoms that do not overlap with the docked active ligands are typically removed first, thus, making the models slimmer (Gen #0 vs Gen #11 in Figure 2C). Likewise, atoms with minimal adverse effects are removed last, which decreases the perceived “ligand-likeness” of the optimized models (Gen #11 vs Gen #23 in Figure 2C; Table S11). Notably, focusing the removals on those atoms that improve the model’s average similarity match with only the actives does not generate higher yields than the BR20-guided BR-NiB (Table S12).

### Effect of Docking Sampling on the Optimization

While there are software- and target-specific variations, BR-NiB generated invariably higher yields than the default docking scoring. BR-NiB was tested with three alternative software on four targets. Every enrichment metric was improved with training and test sets for COX2, RXRα, NEU, and MR using the docking software DOCK and GOLD^37^ (Tables S13–S15). With GLIDE SP,^15,16^ the AUC values of COX2 and RXRα could not be boosted. Notably, BR-NiB produced an even higher enrichment for NEU with GLIDE and for NEU and COX2 with GOLD in comparison to PLANTS. Overall, the ability of BR-NiB to work on an equal footing with multiple docking software was expected based on prior R-NiB results.^18^

**Figure 4:**
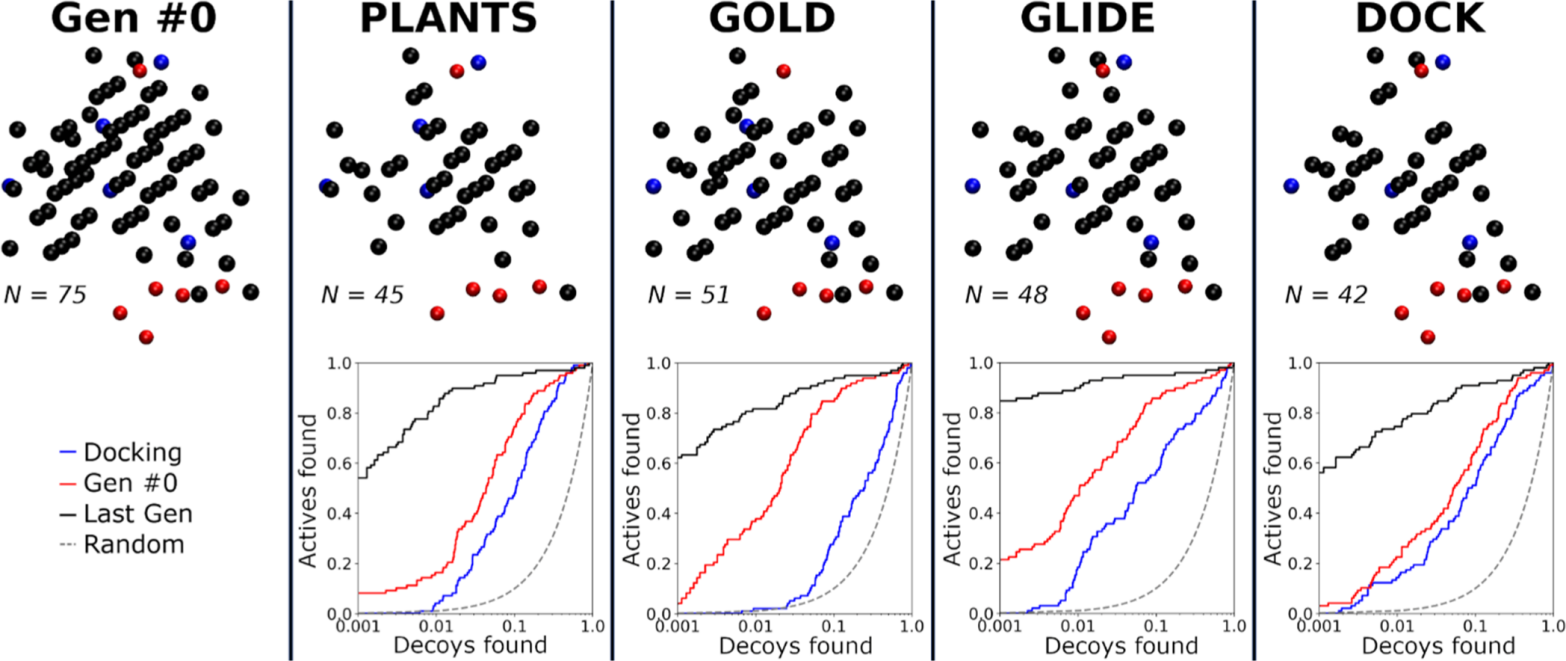
Effect of docking software on the BR-NiB with NEU. The NIB model of NEU binding cavity (NEU; PDB: 1B9V(38)) was optimized using the docking poses of PLANTS, GOLD, GLIDE SP, or DOCK with BR-NiB (Figure 2; Videos S1 and S2). The optimized models are alike; however, the composition differences due to alternative docking sampling affect the ROC curves.

The BR-NiB-optimized models are not merely mirroring the sampling/scoring choices of the docking algorithms, but they focus on core recognition aspects for the active ligands. Different docking software samples and output different binding predictions and, moreover, they can skip compounds (e.g., GLIDE with MR; Table S13). This variation, however small it might be, affects the optimized model composition (Figure 4). Therefore, the cross-use of NIB models, optimized using docking poses from different software, was probed. The limited testing indicates that the optimized models could indeed be cross-used (Table S15). When the PLANTS poses were rescored using the models optimized with the poses of GLIDE, DOCK, or GOLD, all enrichment metrics were improved. This universal applicability of the optimized models is remarkable, when taking a note that the AUC values are at a statistical tie despite the cross-use (Tables 1 vs S15).

### Computing Demands of the Optimization

Individual R-NiB runs are ultra-fast (∼2 to 4 ms/cmpd.),^13^ but the greedy search of BR-NiB can still take long time and plenty of parallel computing (Table S16). The exact demands depend on both the training set size and the input NIB model size. In theory, the optimization can be almost instantaneous, if enough parallel computing resources are committed; but in practice the number of CPUs (or cores) and RAM are the bottlenecks.

BR-NiB was tested using 15 CPUs, when the training sets were divided into 10,000 compound subsets for the parallel execution. In total, the duration of optimization ranged from 2 h, 4 h 43 min to 5 h 47 min for MR, NEU, and COX2, respectively (70:30 ratio in Table S14). The optimization becomes gradually faster when moving closer to the conclusion (e.g., Gen #0: 8 min vs Gen #X: 6 min with MR). PPARγ is a prime example of a demanding target regarding both the cavity size (*N* = 144; Figure S1) and training/test set size (*N* = 25,750; Table S1). The scalability of BR-NiB was probed by repeating the initial steps of PPARγ model optimization using a supercomputer: the doubling of the CPU count from 20 to 40 increased the pace from ∼1.5 to ∼1 h/gen.

If necessary, the BR-NiB iteration can be stopped at an earlier point because, typically, decent enrichment is acquired at the midpoint (Figures S2–S3). Alternatively, the branching could be circumvented by pruning all those atoms whose individual deletion improves the enrichment at one go (Figure S10) and performing the normal BR-NiB after these removals. Although this cut-and-go approach is undeniably fast and it typically improves the enrichment over docking or standard R-NiB (Table S17), it is not on par with the full-scale BR-NiB (Table 1). The cavity atoms and, for better or worse, their removals are interconnected, and hence it pays off to balance their effects and improve the model fitness via successive iterations.

When examining the Gen #1 model variants of RXRα (*N* = 57) in detail, nine cavity atoms, whose individual removals improved the BR20 value the most, were removed also from the fully optimized model (Figure S10). In contrast, nine cavity atoms, whose removal decreased the BR20 value the most in Gen #1, were similarly kept throughout the optimization. Therefore, in this case, the greedy search could have been sped up ∼30% by removing or locking in place these atoms before embarking on a full-scale optimization. This practice would require the careful use of balanced thresholding during the optimization, which is not considered further here.

### Testing on Additional Targets and Different Databases Suggests Wider Applicability

Because the optimization with the random 70:30 training/test set division generated high enrichment (Table 1), five extra DUD-E sets were selected for performing BR-NiB with only this computationally light set up (Tables 2, S1 and S7). The targets, which were selected with additional diversity in mind, included serine/threonine-protein kinase AKT (AKT1), dopamine receptor D3 (DRD3), acetylcholinesterase (ACES), catechol-*o*-methyltransferase (COMT), and focal adhesion kinase 1 (FAK1). The AUC values of docking were improved substantially for all targets except COMT in the testing (Table 2). BR-NiB provided the biggest boost for ACES, as its AUC value improved from 0.39 to 0.69. Likewise, the AUC value of FAK1 improved from 0.80 to 0.95, indicating exceptional ability of the optimized model to increase the docking yield. Importantly, the BR-NiB was able to improve EFd 1% values of docking 3.6-, 36,7-, 2.0-, and 3.7-fold for AKT1, DRD3, COMT, and FAK1, respectively. The results were impressive also for ACES as its EFd 1% value jumped from 0 to 18 in the testing. To sum up, the DUD-E benchmarking (Tables 1 and 2) suggests that BR-NiB works exceptionally well with diverse drug targets.

Testing was also carried out with five DUDE-Z and Extrema benchmarking sets recently introduced by Stein et al.^32^ The sets included NEU, adenosine A_2A_ receptor (A2AAR), ACES, heat shock protein 90 (HSP90), and androgen receptor (AR). The random 70:30 training/test set division results indicate that the BR-NiB method clearly provides an excellent boost for docking performance especially regarding early enrichment with both of the benchmarking databases (Table 3). The success with the Extrema set, which is designed to avoid over-optimization of the electrostatics, is explained by the fact that both R-NiB^13^ and BR-NiB approaches focus heavily on shape similarity and thus, the decoys with extreme charges should not challenge either of them. With DUDE-Z, the most difficult case was the HSP90 set, which has been described to be one of the most demanding targets for the structure-based drug discovery methods.^29^ Here, the AUC was not significantly improved with the BR-NiB method over docking (from 0.52 to 0.56); however, the early enrichment was improved (e.g., EFd 1% from 0.0 to 6.5).

**Table 3:** Test Set Results of Five DUDE-Z and Extrema Sets^a^

set	software/method	yield	NEU	AA2AR	ACES	HSP90	AR
**DUDE-Z****^b^**	docking 70:30	AUC	0.90 ± 0.03	0.75 ± 0.02	0.33 ± 0.02	0.52 ± 0.05	0.60 ± 0.03
		EFd 1%	29.0	27.4	1.4	0.0	1.2
		EFd 5%	58.1	41.5	4.3	3.2	13.3
		BR20	0.53	0.41	0.07	0.05	0.13
	BR-NiB 70:30	AUC	0.95 ± 0.02	***0.84 ± 0.02***	***0.67 ± 0.03***	0.56 ± 0.05	***0.85 ± 0.03***
		EFd 1%	***77.4***	23.8	***9.3***	***6.5***	***21.7***
		EFd 5%	***83.9***	***45.1***	***19.3***	***25.8***	***50.6***
		BR20	***0.84***	***0.43***	***0.24***	***0.19***	***0.44***
	training set ligs/decs		66/4270	352/20,402	300/6690	67/3990	188/9625
	test set ligs/decs		31/1830	164/8742	140/2868	31/1710	83/4123
	docking70:30	AUC	0.80 ± 0.05	0.93 ± 0.01	0.55 ± 0.03	0.47 ± 0.05	0.56 ± 0.03
**Extrema****^c^**		EFd 1%	10.0	46.9	3.7	0.0	0.0
		EFd 5%	13.3	66.2	10.3	3.7	0.0
		BR20	0.13	0.63	0.10	0.03	0.02
	BR-NiB 70:30	AUC	***0.94 ± 0.03***	***0.90 ± 0.02***	***0.66 ± 0.03***	***0.99 ± 0.01***	***0.94 ± 0.02***
		EFd 1%	***63.3***	***62.8***	***14.7***	***92.6***	***48.1***
		EFd 5%	***76.7***	***70.3***	***19.9***	***100.0***	***75.3***
		BR20	***0.72***	***0.67***	***0.19***	***0.92***	***0.66***
	training set ligs/decs		68/48,588	337/94,790	317/65,995	61/76,469	188/98,175
	test set ligs/decs		30/20,823	145/40,769	136/28,281	27/32,775	81/42,072

a The values are shown in bold and italics, if improved in comparison to the docking. Only those AUC values that are over the error margin are highlighted. The testing was performed with the 70:30 training/test set division using equal shape/ESP (0.5/0.5) weight. The Wilcoxon statistic^34^ was used for the AUC error estimation. The PDB codes for the target structures used are the following: 1B9V (NEU), 3EML (AA2AR), 6LTK (HSP90), 2AM9 (AR), and 1 × 10^66^ (ACES).

b The DUDE-Z is the optimized version of the DUD-E set.

c The active compounds from the DUD-E set were used (training/test) in addition to the Extrema decoys.

The BR-NiB performance was also tested with 10 sets from the MUV^31^ database, which has been specifically designed to avoid analogue bias (Table S18). Like the DUD database, MUV also contains too few active ligands for building robust training/test set divisions. Both the default docking and BR-NiB generated low AUC values, which reflects the fact that the sets contain only a few active compounds that represent different chemotypes. As mainly intended for ligand-based approaches, the MUV sets are not optimal for docking benchmarking, that is, no reference protein structures are given, and the included compounds may have alternative binding sites. Regardless, BR-NiB was able to generate decent or even excellent early enrichment values, if compared to docking (Table S18). With Rho kinase 2, both the AUC and EFd 1% values were improved from 0.44 ± 0.05 and 3.3, respectively, to 0.71 ± 0.05 and 33.3. Likewise, with human immunodeficiency virus or HIV set, the corresponding values improved from 0.45 ± 0.05 and 3.3, respectively, to 0.69 ± 0.05 and 23.3.

### Validation: Very Early Enrichment Improvement Using the Preoptimized Models

BR-NiB is not aiming to recognize correct binding poses but only to separate actives from inactives.^13,18^ Irrespective, the limited root-mean-square deviation (RMSD) analysis with co-crystals (1–3 Å ranges in Table S18) indicates that neither BR-NiB nor R-NiB steer the selection toward “wrong” poses, if compared to the default docking scoring. A better measure of success is the hit-rate in demanding benchmarking tests involving multiple targets. Thus, as a further validation, the BR-NiB-optimized models were tested using new benchmarking sets for MR and NEU, which had accumulated enough additional data since the generation of their DUD-E sets. The aim was to emulate the extreme difficulty of virtual screening campaigning, in which only minute amounts of active ligands are buried in a vast and diverse library composed of drug-like compounds. The problems in building unbiased test sets are well-documented;^39−41^ thus, this validation step is intended to act only as an extra check for the new method’s effectiveness. The included decoy compounds are not property matched with the known actives nor experimentally verified (not typical with other benchmarking sets either), but the massive difference in the compound numbers should lean heavily in favor of finding decoys rather than active ligands by chance. Here, the rescoring results are directly comparable only against the original docking algorithm.

Because high potency levels are typically reached only via the optimization of low potency hits, three sets were generated for both targets with varying potency levels. The validation sets included 0.014% of “high potency” (IC_50_ < 1 μM), “mid-to-low potency” (IC_50_ = 1–50 μM), or “high-to-low potency” (IC_50_ = < 50 μM) active ligands buried into a large library. Overall, the validation results (Table 4) were similar to the test set results (Table 1), suggesting that the BR-NiB offers far better enrichment than the docking scoring. A direct comparison between the testing and validation results is skewed because the latter sets contain undoubtedly considerably more inactive compounds in the decoy set (MR: 1.8% vs 0.014%; NEU: 1.6% vs 0.014%). Regardless, the AUC values showed that the BR-NiB-optimized models worked at a comparable level with both sets. Even when focusing at the EFd 1% values, BR-NiB fared only slightly better in the testing than during the validation (Tables 1 vs 4).

**Table 4:** Docking and Brute Force Negative Image-Based Rescoring with Validation Sets^a^

		docking			BR-NiB-guided rescoring		
target	yield	IC_50_ < 1 μM	IC_50_ < 50 μM	IC_50_ 1–50 μM	IC_50_ < 1 μM	IC_50_ < 50 μM	IC_50_ 1–50 μM
MR	AUC	0.37 ± 0.07	0.44 ± 0.06	0.63 ± 0.07	***0.84* ± *0.06***	***0.88* ± *0.05***	***0.87* ± *0.05***
	EFd 0.1%	0	0	0	***5***	***15***	***5***
	EFd 0.5%	0	5	0	***15***	***15***	***5***
	EFd 1%	0	10	5	***30***	***20***	***5***
	EFd 5%	5	10	5	***35***	***35***	***45***
NEU	AUC	**0.96 ± 0.03**	**0.95 ± 0.03**	**0.95 ± 0.03**	**0.97 ± 0.03**	**0.95 ± 0.03**	**0.94 ± 0.04**
	EFd 0.1%	0	5	5	**35**	**15**	**10**
	EFd 0.5%	15	10	5	**55**	**40**	**35**
	EFd 1%	20	15	15	**65**	**55**	**50**
	EFd 5%	65	70	70	**85**	**75**	**75**

a Docking with default scoring (PLANTS) and BR-NiB-guided rescoring (SHAEP) were tested with three validation sets: “*high potency*” (IC_50_ ≤ 1 μM), “*low-to-mid potency*” (IC_50_ = 1–50 μM), or “*high-to-low potency*” (IC_50_ = ≤ 50 μM) for MR and NEU. The active ligands (*N* = 20) were mixed into the SPECS library (*N* = 140,626), making the active compound concentration 0.014% for the validation sets. The best results for each potency level are shown in bold and italics. The Wilcoxon statistic^34^ was used for the AUC error estimation.

Importantly, by testing just 0.1% (∼140 cmpd.) or 0.5% (∼700 cmpd.) of top-ranked compounds, the screening hit-rates would have been at least 5–15% for MR and 10–55% for NEU (Table 4). The enrichment improvement was highest for the “high potency set”, but the upward trend was also clear for the less potent sets that exemplify better the non-optimized screening hits. When this is translated to hit numbers, the excellence of BR-NiB becomes apparent. With MR, BR-NiB-guided screening would have found 1–3 hits, whereas the default docking scoring would have found hardly any. With NEU, the BR-NiB-guided selection would have performed even better, by recognizing 2–7 or 6–11 hits as opposed to 1–3 found by the default scoring.

## Discussion

Target-tailored rescoring methods are frequently needed for improved molecular docking yields in virtual screening.^42^ Herein, we report a BR-NiB (Figure 2; Videos S1 and S2) method that augments the composition of protein cavity or NIB models (Figure S1; Table S10) for improved docking rescoring performance. The NIB models, which are used in a shape/ESP similarity comparison with the flexible docking poses, are subjected to iterative atom removals and benchmarking (Figure 2). In the negative image-based rescoring (Figure 1), the BR-NiB-optimized models boost docking massively, that is, the active ligands are effectively separated from the decoys. The effectiveness of BR-NiB was verified by rigorous testing with multiple targets/sets (Tables 1–3, S5 and S18) and with demanding validation sets for two targets (MR and NEU in Table 4).

BR-NiB (Figure 2; Video S2) is a hybrid method that builds on the strengths of both ligand- and structure-based drug discovery methods. Therefore, the biggest hurdle of BR-NiB is the need for both the protein 3D structure and compound training set with validated activity information (Table S1). Regardless, the training set requirements of BR-NiB are moderate and, thus, the method should be well-suited for early-stage drug discovery projects, where there is a limited amount of compound data available. While the optimization can take a lot of time depending on a target, the process can easily be sped up by extra parallelization or, in theory, via the careful use of thresholding (Table S16). Finally, despite the automation, the input NIB models must be generated beforehand, and, likewise, the training set used in the optimization must be docked separately. However, because the atomic composition is optimized, the input model obviously does not have to be perfect to begin with and, moreover, the method is not docking software-specific (Tables S13–S15; Figure 4) nor big data-driven (Table S1).

BR-NiB reminds regular pharmacophore (PHA) modeling, which can work excellently in virtual screening by focusing on a few key ligand descriptors with or without the added protein “exclusion zone” information.^43^ Similar to BR-NiB, it is also possible to use docking poses as the pre-aligned conformation database to perform the PHA screen. Because both the active ligands and “inactive” decoys influence the final NIB model composition, BR-NiB could conceptually be perceived even as shape/ESP-based or shape-focused PHA modeling. Additionally, BR-NiB bears resemblance to field-based quantitative structure-activity relationship (F-QSAR) and even machine learning techniques, but there are major differences. In practice, the most predictive F-QSAR models require focusing on highly specific compound series, prefiltered before the model building, and, furthermore, the model’s connection to the protein structure can be elusive. Machine learning techniques^24−27^ can have limited usability due to the massive data requirements and, frankly, their “black box” logic can also be a cause of unease. A direct comparison to these established methods is moot because BR-NiB is exclusively a rescoring method and, thus, tied to the limitations of docking sampling. Regardless, in BR-NiB, both the cavity model and the underlying docking poses are always tightly connected to the protein (Figure 1), data requirements are moderate (Table S1), and the optimization steps can be easily backtracked (Figure 2).

There is plenty of room for improvements in the implementation of BR-NiB. Establishing the best practices for the optimization require yet more testing and experimental validation—an iterative process that is outside the scope of this singular study. However, the fact that BR-NiB ignores the possible target protein flexibility or ligand binding induced-fit effects is likely to be a very persistent problem. This is seen for example with PPARγ, when the BR-NiB results improved noticeably, when using an alternative protein structure for the docking and input NIB model generation (Table 2). A potential workaround is to generate multiple NIB models for different protein structures or even combine and optimize multiple models simultaneously (e.g., PDE5; Figure 3; Tables 1 and S3); however, this sort of approach is computationally costly and requires extra effort from the user. To be fair, this protein conformation problem is inherent to using molecular docking as a high-throughput virtual screening method in the first place.^44,45^

Because the training and testing are both showing similar enrichment boost to docking (Tables 1 vs S6 or Tables 2 vs S7), there is no serious concern that the BR-NiB method is overfitting. Regardless, it is likely that the NIB models become more focused on certain active ligand chemotypes present in the training set than others during the optimization. While this specialization should at least in theory lower the diversity or perceived novelty of the eventual virtual screening hits, BR-NiB did slightly better than the default docking in the similarity analysis with the top-ranked validation set compounds (data not shown). One could argue that this phenomenon is commonplace irrespective of the applied method when working with limited training sets. However, even without considering the induced-fit effects, it is not possible that a singular NIB model could be a match for all active ligands present in a truly diverse training set. The optimization is a give-and-take process that makes greedy choices based on the target enrichment metric improvement (Figure 2C). Paradoxically, a potential workaround could be to train multiple NIB models with purposefully different sets and apply the alternatively trained models to the virtual screening. Regardless, the BR-NiB testing should continue with alternative benchmarking sets and different databases in the future. Testing with the MUV database^31^ (Table S18), DUDE-Z, Extrema (Table 3) and the newly made validation sets (Table 4) shows that BR-NiB works also with other benchmarking databases than DUD or DUD-E (Tables S5, 1 and 2). Ultimately, the method’s practical usability must be confirmed in actual screening usage that includes *in vitro* validation.

Access to effective drugs and healthcare is a major obstacle for the equal prospects of wellbeing in the world, and the unsustainably high drug prices is a big contributor to this severe problem even in the developed Western countries.^46^ Inarguably, it is a multilayered problem involving prohibitive costs related to regulation and safety, but the risk-aversion and profit-driven tactics are also to blame.^47^ Computer-aided drug discovery, especially virtual screening methods, provide the means to lower the development costs and, importantly, open the door also for academic and open-access efforts by non-profit institutions.^48^ Thus, it is noteworthy that BR-NiB can be performed with free software, the BR-NiB code itself is freely downloadable under MIT license via GitHub (https://github.com/jvlehtonen/brutenib) and, in most cases, the optimization can be done using a regular desktop or even a laptop computer within a reasonable amount of time (Table S16).

In summary, a new rescoring methodology, BR-NiB, is presented for improving docking enrichment to a level that facilitates effective drug discovery in virtual screening campaigning.

## Experimental Section

### Protein and Ligand Structure Preparation for Benchmarking

The protein X-ray crystal structures were acquired from the Protein Data Bank (PDB; Table S1)^49^ for the molecular docking and cavity detection or filling. The 3D structure editing and PDB-to-MOL2 conversion was done using BODIL.^50^ The structures were protonated to match pH 7.4 using REDUCE3.24.^51^ No crystal waters were considered during the docking or cavity detection.

The ligand sets (Table S1) for 12 drug targets, including cyclooxygenase-2 (COX2), retinoid X receptor alpha (RXRα), MR, NEU, PDE5, ER, and peroxisome proliferator-activated receptor gamma (PPARγ), serine/threonine-protein kinase AKT (AKT1), dopamine receptor D3 (DRD3), catechol-*o*-methyltransferase (COMT), acetylcholinesterase (ACES), and focal adhesion kinase (FAK1), were acquired from the DUD-E database.^29^ Likewise, seven equivalent sets were also acquired from the DUD^30^ database, if available, for limited testing (Table S5).

Five DUDE-Z and five Extrema benchmarking sets were also acquired for testing^32^ (Table 3). DUDE-Z is described to be an optimized version of the DUD-E database. In the DUDE-Z design, special attention has been given to the charge matching between the active and decoy compounds. The Extrema design consist of decoys with extreme charges to prevent over-optimization based on the electrostatic interactions. With the Extrema, the active compounds in the corresponding DUD-E sets were used as the active molecules.

Altogether, 10 test sets were acquired from the MUV database^31^ for the BR-NiB testing (Table S18). Because the database is designed mainly for ligand-based virtual screening, MUV aims to minimize the analogue bias by paying special attention to the spatial randomness, and it contains only 1.16 compounds per scaffold class. Each one of the 17 sets contain only 30 active ligands and 15,000 decoy molecules. If a suitable target protein 3D structure with a valid co-crystal ligand was available in the PDB, the MUV set in question was also tested. Although no inhibitor-bound X-ray crystal structure was available for the ephrin type-A receptor 4 set, the same binding cavity was used for docking as in a prior study by Gu et al.^52^ The dopamine receptor D1 set was excluded because it included allosteric modulators that could bind to multiple different allosteric sites.^53^

Prior to the flexible docking, the ligands were translated from the simplified molecular-input line-entry system (SMILES) strings to the 3D SYBYL MOL2 format, protonated at pH 7.4, alternative tautomeric states were generated and, finally, OPLS3 partial charges were incorporated using LIGPREP and MOL2CONVERT in MAESTRO 2017-1 (Schrödinger, LLC, New York, NY, USA, 2017). The active/decoy compounds of the DUD-E sets were also divided randomly into training (70 or 10%) and test (30 or 90%) sets (Table S1) for unbiased testing (Figure 2). The random shuffling was done using standard C++ library Mersenne Twister 19937 pseudo-random number generator.^54^

### Flexible Molecular Docking

PLANTS and its default scoring function ChemPLP was used as the primary docking algorithm due to its proven effectiveness with R-NiB;^18^ however, limited testing was additionally done using GLIDE 2018-1^15,16^ (MAESTRO 2018-1, Schrödinger, LLC, New York, NY, USA, 2018) at standard precision (SP), DOCK 6.8,^14^ and GOLD 5.6.3.^37^ If available, the co-crystallized ligands (Figure 1B; Table S1) were used as the docking centroids with a search radius of 10 Å. Particularly, some MUV targets lacked the co-crystallized ligand data, so the centroid was set inside the putative ligand binding-cavity. The relevant docking settings are detailed in prior studies.^13,18^

### Negative Image-Based Model Generation

The cavity-based NIB models (Figure S1) were generated using default PANTHER 0.18.15,^19^ but parameters such as the *packing method*, *ligand distance limit*, and/or *box radius* were adjusted, if necessary. If possible, the cavity centroids were taken from the co-crystallized ligands (Table S1; Figure S1). With PDE5, the NIB models could also be combined to generate a hybrid model (Figure 3). It was verified visually that the NIB models roughly covered the cavity volume involved in the known ligand binding. The input NIB models are given in the Supporting Information, and the PANTHER settings for DUD and DUD-E are explained in prior studies.^13,18,22^

### Negative Image-Based Rescoring

In negative image-based rescoring (R-NiB; Figure 1),^13^ the shape/electrostatics of flexible docking poses is compared against the cavity-based NIB model using the similarity comparison algorithm SHAEP.^21^ The implementation of the *--noOptimization* option ensures that the poses are not realigned during the rescoring. The similarity comparison was performed with either the default 50/50 ratio of the shape/ESP or only the shape score. The performance was not tested with only the ESP score because typically the shape is in a dominant role in the SHAEP scoring. The R-NiB protocol is explained thoroughly at the practical level in a prior study.^22^ Likewise, ligand-based rescoring was performed by comparing the co-crystallized ligands (Figure S1; Table S1) against the docking poses without realignment.

### Brute Force Negative Image-Based Optimization

In the BR-NiB (Figure 2; Videos S1 and S2), the effect of each cavity atom in the NIB model is evaluated iteratively and automatically. Although a true exhaustive search would be guaranteed to generate a global optimum model, whereas a greedy search is more likely to stop at a local optimum, the calculations would be too time-consuming with a brute force search. If the input model contains 50 cavity atoms (*a*_*n*_) and its optimal size would be 40 atoms (*a*_1_), a true exhaustive search would run all possible 1.3e10 test cases before concluding (eq 1), while the greedy search would need only 495 (eq 2).
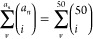
1
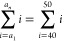
2

BR-NiB is performed using a template of the NIB model from which cavity atoms are removed one at a time before the individual R-NiB runs. The iterative editing, rescoring, and enrichment evaluation processes are performed using a specific target enrichment metric such as the EFd, AUC, and BEDROC^33^ with alpha value 20 (BR20) using ROCKER 0.1.4.^55^ Note that the optimal BR value is dependent on the size of the training set; but it was not altered here to keep the results comparable. The model producing the best performance for each generation is put forth into the next cycle of editing and rescoring. The greedy search is deterministic and, thus, all things being equal, it should always lead to the same model composition. For this same reason, it is also unlikely to produce global optimum similarly as a true exhaustive search would; however, BR-NiB boosts docking with a sensible amount of computing. The iteration ends when the atom removals stop improving the target metric (Figure 2; Video S1). The final BR-NiB-optimized NIB models are given in the Supporting Information. The BR-NiB code, needed for performing the optimization automatically, is provided online for free academic or commercial use under MIT license via GitHub (https://github.com/jvlehtonen/brutenib).

### Validation: Benchmarking against a Massive Drug-like Compound Library

New validation sets were generated for MR and NEU with varying activity ranges. In the “high potency set” were included active ligands with an IC_50_ value of <1 μM, whereas in the “mid-to-low potency set” or “high-to-low potency set” were included ligands with the IC_50_ range of 1–50 μM or <50 μM, respectively. The active ligands were taken randomly from the ChEMBL database. Duplicates with the original DUD-E sets were avoided and, furthermore, it was checked that the active ligands could fit into the binding pockets. Molecules with a MW of over 550 g/mol were excluded from the data sets to ensure that the molecules were not too complicated for the docking software. In practice, 20 randomly selected active ligands of each potency category (<1 μM, < 50 μM and 1–50 μM) were mixed with the drug-like small-molecule compounds of the SPECS database (acquired 08/2020; *N* = 140,626; MW of over 550 g/mol and rotatable bond number of >8 excluded to include only the most ligand-like molecules), making the verified active ligand concentration 0.014% for each validation set (at least 1.5% for the DUD-E sets; Table S1). Finally, all compounds were prepared with LIGPREP, docked with PLANTS, and rescored with SHAEP using the BR-NiB-optimized NIB models similarly as described for the DUD-E sets.

### Data Analysis and Figure Preparation

Figures were prepared using BODIL^50^ and VMD 1.9.2.^56^ ROCKER^55^ was used to plot the ROC curves. The AUC, BR20, and EFd values were calculated using ROCKER. The Wilcoxon statistic^34^ estimates the standard deviation in the AUC calculations. The EFd 0.1, 0.5, 1, and 5% values correspond to the percentage of true positive ligands found when 0.1, 0.5, 1, or 5% of “inactive” decoy compounds have been discovered. The compounds that were skipped during docking were added to the bottom of the results in the order that corresponds random picking to make the early enrichment results comparable. The RMSD values were calculated for those DUD-E compounds with both docking poses and co-crystallized ligand conformers using rmsd.py (MAESTRO 2018-1). The conformers were aligned in the 3D space using the protein backbone C^α^ atoms using VERTAA in BODIL.^50^ The co-crystallized ligands (Table S1), which were prepared similarly as the DUD-E compounds but without allowing a heavy atom realignment, were taken from the PDB entries with the highest available resolution.

## Data and Software Availability

BR-NiB code is available under MIT license via GitHub (https://github.com/jvlehtonen/brutenib). All the data needed for repeating the experiments, including input NIB models, BR-NiB-optimized models, benchmarking sets, random training/test set divisions, and specific validation sets of NEU and MR, are available in the Supporting Information (zip file). The original DUD-E (http://dude.docking.org) sets, DUD sets (http://dud.docking.org/), MUV sets (https://www.tu-braunschweig.de/en/pharmchem/forschung/baumann/translate-to-english-muv), DUDE-Z and Extrema sets (https://dudez.docking.org/), PDB (https://www.rcsb.org/) entries, SPECS (www.specs.net) library, and ChEMBL (http://dude.docking.org) library are available online. BODIL (http://users.abo.fi/bodil/about.php), REDUCE (http://kinemage.biochem.duke.edu/software/reduce.php), PANTHER (http://www.medchem.fi/panther/), ROCKER (http://www.medchem.fi/rocker/), SHAEP (http://users.abo.fi/mivainio/shaep/download.php), PLANTS (http://www.tcd.uni-konstanz.de/plants_download/), DOCK (http://dock.compbio.ucsf.edu/Online_Licensing/index.htm), GOLD (https://www.ccdc.cam.ac.uk/solutions/csd-discovery/Components/Gold/), and LIGPREP, MOL2CONVERT, and GLIDE in MAESTRO (https://www.schrodinger.com) are available online for downloading. MAESTRO is a commercial modeling package, but it can be acquired free for academic usage. GOLD is not freely available for academic usage, but academic institutions are applicable to substantial discount.

## Supporting Information Available

Target structures and benchmarking sets, Gen #0 training set results, PDE5 model results, effect of target metric on optimization, BR-NiB training set enrichments, BR-NiB training/test set 70:100 and 10:100 division enrichment results, ligand-based rescoring results with the X-ray co-crystals, NIB model compositions, shape fitting results, BR-NiB results with alternative docking software, docking cross-use results with the BR-NiB-optimized NIB models, computing demands of the optimization, single-generation optimization results, enrichment metrics of the MUV set, RMSD results of BR-NiB-selected poses, BR-NiB enrichment evolution over optimization generations, ROC curves, effect of cavity atom removals, and references (PDF)
Initial NIB models, BR-NiB-optimized models, compositions of the benchmarking, and validation sets (ZIP)
BR-NiB optimization (MOV)
BR-NiB generation #0 vs generation #49 (MOV)

## Supplementary Material

ci1c01145_si_002.zip

ci1c01145_si_003.mov

ci1c01145_si_004.mov
