# Supplementary material for: Optimization of Cavity-Based Negative Images to Boost Docking Enrichment in Virtual Screening
Source: J Chem Inf Model. 2022 Feb 8;62(4):1100–12. doi: 10.1021/acs.jcim.1c01145 (PMC8889583; doi:10.1021/acs.jcim.1c01145)
Supplement: Supplementary file 1 — ci1c01145_si_001.pdf [file ci1c01145_si_001.pdf]

Supporting Information for

Optimization of Cavity-Based Negative Images to Boost Docking Enrichment in Virtual Screening

Sami T. Kurkinen[1,2,3], Jukka V. Lehtonen[4,5], Olli T. Pentikäinen[1,2,3], Pekka A. Postila[1,2,3,*]

[1] Institute of Biomedicine, Integrative Physiology and Pharmacy, FI-20014 University of Turku, Turku, Finland.

[2] Aurlide Ltd., FI-21420 Lieto, Finland.

[3] InFLAMES Research Flagship Center, FI-20014 University of Turku, Turku, Finland.

[4] Structural Bioinformatics Laboratory, Biochemistry, Faculty of Science and Engineering, FI-20500 Åbo Akademi University, Turku, Finland.

[5] InFLAMES Research Flagship Center, FI-20500 Åbo Akademi University, Turku, Finland.

[*]Corresponding author: pekka.postila@utu.fi

Table S1. Target protein structures and DUD-E small-molecule benchmark sets.

Target protein [(1)]	PDB code	Resolution (Å)	Mechanism of action	Train/ test [(5)]	No. of active ligands [(4)]	No. of inactive decoys [(5,6)]	Active ligands concentration (%)
COX2	3LN1	2.40	inhibitor	100:100<br>70:30<br>10:90	435/0<br>307/128<br>61/374	23,136/0<br>16,195/6,941<br>2,314/20,822	1.9<br>1.9/1.8<br>2.6/1.8
RXRα	1MV9	1.90	agonist	100:100<br>70:30<br>10:90	131/0<br>91/40<br>13/118	6935/0<br>4855/2,080<br>694/6,241	1.9<br>1.9/1.9<br>1.9/1.9
MR	2AA2	1.95	agonist	100:100<br>70:30<br>10:90	94/0<br>65/29<br>9/85	5146/0<br>3602/1,544<br>515/4,631	1.8<br>1.8/1.9<br>1.7/1.8
NEU	1B9V	2.35	inhibitor	100:100<br>70:30<br>10:90	98/0<br>68/30<br>9/89	6197/0<br>4338/1,859<br>620/5,577	1.6<br>1.6/1.6<br>1.5/1.6
PDE5	1UDT / 1XOZ[(2)]	2.30 / 1.37	inhibitor	100:100<br>70:30<br>10:90	398/0<br>278/120<br>39/359	27,520/0<br>19,264/8,256<br>2,752/24,768	1.4<br>1.4/1.5<br>1.4/1.4
ER	1SJ0	1.90	agonist/ antagonist	100:100<br>70:30<br>10:90	383/0<br>273/110<br>38/345	20,663/0<br>14,464/6,199<br>2,066/18,597	1.9<br>1.9/1.8<br>1.8/1.9
PPARγ	2GTK	2.10	co-agonist	100:100<br>70:30<br>10:90	484/0<br>338/146<br>48/436	25,256/0<br>17,679/7,577<br>2,526/22,730	1.9<br>1.9/1.9<br>1.9/1.9
AKT1	3CQW	2.00	inhibitor	70:30	410/88	11,501/4,934	3.6/1.8
DRD3	3PBL	2.89	antagonist	70:30	336/144	23,820/10,214	1.4/1.4
COMT	3BWM	1.98	inhibitor	70:30	28/13	2,694/1,155	1.0/1.1
ACES	1E66	2.10	inhibitor	70:30	317/136	18,365/7,873	1.7/1.7
FAK1	3BZ3	2.20	inhibitor	70:30	70/30	3,745/1,605	1.9/1.9

(1) Target proteins: cyclooxygenase 2 (COX2)[1], retinoid X receptor alpha (RXRα)[2], mineralocorticoid receptor (MR)[3], neuraminidase (NEU)[4], phosphodiesterase 5 (PDE5)[5,6], estrogen receptor (ER)[7], peroxisome proliferator-activated receptor gamma (PPARγ)[8], serine/threonine-protein kinase (AKT1)[9], dopamine receptor D3 (DRD3)[10], catechol-o-methyltransferase (COMT)[11], acetylcholinesterase (ACES)[12] and focal adhesion kinase 1 (FAK1)[13].
(2) Only PDB entry 1UDT[5] was used for the docking; however, both 1UDT and 1XOZ[6] were used in the negative image-based (NIB) model generation.
(3) Train/test set ratios (100:100, 70:30, 10:90): the percentage of ligands used in the training (100 %, 70 %, 10 %) in relation to the percentage used in the testing (100 %, 30 %, 90 %).
(4) The number (No.) of DUD-E (A Database of Useful (Docking) Decoys –Enhanced) active ligands, inactive decoy molecules after performing the ligand preparation with LIGPREP in MAESTRO (status before the docking screening). The source of the used benchmark test sets was DUD-E (A Database of Useful (Docking) Decoys –Enhanced)[14].
(5) The actual decoy numbers in train/test sets might vary with couple molecules because the original DUD-E decoy sets contain duplicates, and during molecules conversion with LIGPREP the software might skip some molecules.

Table S2. Improving docking performance for six targets using negative image-based rescoring.

Train/ test (1)	Method(2)	Yield	COX2	RXRα	MR	NEU	ER	PPARγ
100:100	BR-NiB / Gen #0	AUC	***0.76±0.01***	***0.95±0.01***	***0.80±0.03***	***0.90±0.02***	0.64±0.02	0.65±0.01
		EFd 1%	***18.2***	***56.5***	***5.3***	***16.3***	***25.3***	0.4
		EFd 5%	***40.5***	***85.5***	***22.3***	***54.1***	***39.4***	7.6
		BR20	***0.39***	***0.76***	***0.24***	***0.47***	***0.38***	0.09
	BR-NiB / Gen #0 + shape only	AUC	***0.79±0.01***	0.77±0.02	***0.72±0.03***	***0.90±0.02***	0.67±0.02	0.75±0.01
		EFd 1%	***17.0***	***12.2***	***13.8***	***10.2***	18.0	9.3
		EFd 5%	***39.1***	29.0	***31.9***	***53.1***	33.1	30.8
		BR20	***0.38***	0.28	***0.30***	***0.42***	0.33	0.29
70:30	BR-NiB / Gen #0	AUC	***0.76±0.02***	***0.95±0.02***	***0.78±0.03***	0.90±0.02	0.62±0.02	0.65±0.02
		EFd 1%	***17.9***	***53.8***	***3.1***	***16.2***	25.6	0.0
		EFd 5%	***41.0***	***84.6***	***15.4***	***54.4***	36.3	6.8
		BR20	***0.39***	***0.74***	0.19	***0.47***	0.36	0.09
70:30	BR-NiB / Gen #0	AUC	***0.76±0.02***	***0.95±0.02***	***0.84±0.05***	0.90±0.04	0.70±0.03	0.64±0.02
		EFd 1%	***16.4***	***57.5***	***10.3***	***16.7***	24.5	1.4
		EFd 5%	***42.2***	***87.5***	***37.9***	***60.0***	47.3	9.6
		BR20	***0.39***	***0.78***	***0.36***	***0.48***	0.44	0.10
70:30	BR-NiB / Gen #0 + shape only	AUC	***0.79±0.02***	0.76±0.03	***0.71±0.04***	0.90±0.03	0.65±0.02	0.75±0.02
		EFd 1%	***16.6***	***12.1***	***10.8***	***8.8***	17.6	10.7
		EFd 5%	***38.8***	28.6	***29.2***	***51.5***	31.9	29.9
		BR20	***0.38***	0.27	***0.26***	***0.41***	0.31	0.29
70:30	BR-NiB / Gen #0 + shape only	AUC	***0.79±0.02***	0.79±0.04	***0.75±0.05***	0.90±0.04	0.71±0.03	0.75±0.02
		EFd 1%	***18.0***	***12.5***	***20.7***	***13.3***	18.2	6.2
		EFd 5%	***38.3***	27.5	***37.9***	***56.7***	***36.4***	32.2
		BR20	***0.38***	0.30	***0.39***	***0.45***	***0.36***	0.28
10:90	BR-NiB / Gen #0	AUC	***0.74±0.04***	***0.91±0.06***	***0.81±0.9***	***0.93±0.06***	0.69±0.05	0.65±0.04
		EFd 1%	***14.6***	***53.8***	***11.1***	***33.3***	***28.9***	0.0
		EFd 5%	***32.8***	***84.6***	***22.2***	***66.7***	***47.4***	8.3
		BR20	***0.35***	***0.72***	***0.25***	***0.60***	***0.44***	0.09
10:90	BR-NiB / Gen #0	AUC	***0.76±0.01***	***0.96±0.01***	***0.80±0.03***	***0.90±0.02***	0.64±0.02	0.65±0.01
		EFd 1%	***17.9***	***56.8***	***4.7***	***14.6***	***24.9***	0.5
		EFd 5%	***41.4***	***85.6***	***22.4***	***53.9***	***38.6***	7.6
		BR20	***0.40***	***0.76***	***0.24***	***0.46***	***0.38***	0.09
10:90	BR-NiB / Gen #0 + shape only	AUC	***0.78±0.04***	0.70±0.08	***0.74±0.10***	0.91±0.07	0.72±0.04	0.75±0.04
		EFd 1%	***18.0***	0.0	***11.1***	***11.1***	***23.7***	10.4
		EFd 5%	***32.8***	15.4	***44.4***	***66.7***	***31.6***	33.3
		BR20	***0.36***	0.13	***0.34***	***0.51***	***0.35***	0.33
10:90	BR-NiB / Gen #0 + shape only	AUC	***0.79±0.01***	0.78±0.03	***0.72±0.03***	***0.90±0.02***	0.66±0.02	0.75±0.01
		EFd 1%	***16.8***	13.6	***14.1***	***10.1***	17.7	9.4
		EFd 5%	***39.8***	29.7	***30.6***	***51.7***	33.0	30.0
		BR20	***0.38***	0.30	***0.29***	***0.41***	0.32	0.29

The values are shown in bold and italics, if improved in comparison to the docking of each set (70 %, 30 %, 10 % or 90 %). Only those AUC values that are outside the error margin are highlighted. The Wilcoxon statistic[15] was used for the AUC error estimation.

(1) Training/test set ratios (100:100, 70:30, 10:90): the percentage of ligands used in the training (100 %, 70 %, 10 %) in relation to the percentage used in the testing (100 %, 30 %, 90 %). The relevant train/test set percentage is underlined in the Ratio column.

(2) Methods: brute force negative image-based optimization (BR-NiB; Figure 2) for generation #0 (or Gen #0) either with the equal shape/ESP (0.5/0.5) weight or the shape only (1.0/0.0). Given that no NIB model optimization has been performed yet at Gen #0 of BR-NiB, the results are derived from default negative image-based rescoring (R-NiB; Figure 1).

Table S3. Improving docking rescoring performance for phosphodiesterase-5 using brute force negative image-based optimization.

**Method**(1)	**Yield**	**Model I**(2)	**Model II**(2)	**Combined**(2)
BR-NiB / Gen #0	AUC	0.62±0.02	0.70±0.01	0.65±0.02
	EFd 1%	6.8	8.5	2.5
	EFd 5%	15.8	16.1	9.0
	BR20	0.16	0.18	0.11
BR-NiB	AUC	0.67±0.02	0.62±0.02	***0.82±0.01***
	EFd 1%	11.1	***15.1***	***20.1***
	EFd 5%	22.4	28.1	***41.0***
	BR20	0.21	0.27	***0.39***
BR-NiB / Gen #0 + shape only	AUC	0.63±0.02	0.73±0.01	0.71±0.01
	EFd 1%	6.5	7.8	1.8
	EFd 5%	12.3	19.1	13.6
	BR20	0.14	0.20	0.15
BR-NiB + shape only	AUC	0.72±0.01	0.74±0.01	***0.87±0.01***
	EFd 1%	***15.1***	***13.3***	***27.6***
	EFd 5%	***29.1***	***29.4***	***50.8***
	BR20	***0.29***	0.28	***0.46***

The rescoring values are shown in bold and italics, if improved in comparison to the docking (Table 1). Only those AUC values that are outside the error margin are highlighted. The best results are underlined. The Wilcoxon statistic[15] was used for the AUC error estimation.

(1) Methods: flexible PLANTS[16] docking and brute force negative image-based optimization (BR-NiB; Figure 2) either with the equal shape/electrostatic potential (0.5/0.5) weight or the shape only (1.0/0.0).

(2) The sildenafil- or tadalafil-bound X-ray structures (PDB: 1UDT, 1XOZ)[5,6] were used to generate input Model I and Model II, respectively. The models were also fused together (Combined) for the BR-NiB optimization.

All active ligands and inactive decoys (Table S1) were used in BR-NiB; *i.e.* the train/test set ratio of 100/0 was applied.

Table S4. The effect of target metric on the brute force negative image-based optimization.

Target protein	Target metric	No. of cavity atoms at Gen #0	No. of Gens	AUC	EFd 1%	EFd 5%	BR20
COX2	AUC	44	11	***0.79±0.01***	29.2	***53.3***	0.49
	EFd 1%		9	***0.77±0.01***	***35.9***	49.2	0.49
	BR20		12	***0.78±0.01***	32.2	52.2	***0.50***
RXRα	AUC	56	24	***0.98±0.01***	***78.6***	90.8	***0.87***
	EFd 1%		6	***0.96±0.01***	67.9	88.5	0.79
	BR20		23	***0.97±0.01***	77.9	***91.6***	***0.87***
NEU	AUC	79	25	***0.97±0.01***	73.5	87.7	0.83
	EFd 1%		10	0.93±0.02	49.0	68.4	0.64
	BR20		34	***0.97±0.01***	***82.7***	***91.8***	***0.89***
MR	AUC	57	16	***0.86±0.02***	18.1	48.9	0.42
	EFd 1%		2	0.70±0.03	18.1	28.7	0.29
	BR20		17	0.76±0.03	***33.0***	***48.9***	***0.49***

The best results are shown in bold and italics. Only those AUC values that are outside the error margin are highlighted. All active ligands and inactive decoys (Table S1) were used in the brute force negative image-based optimization (BR-NiB; Figure 2); *i.e.* the training/test set ratio of 100:100 was applied. The Wilcoxon statistic[15] was used for the AUC error estimation.

Table S5 Docking and brute force negative image-based rescoring performance with the DUD sets.

Train/test	Method [(2)]	Yield	COX2	RXRα	MR	PDE5	ER_{ag}	ER_{antag}	PPARγ
100:100	Docking	AUC	0.81±0.01	0.78±0.06	0.80±0.07	0.71±0.04	0.81±0.03	0.81±0.04	0.95±0.02
		EFd 1%	13.5	5.0	26.7	13.7	17.9	15.4	69.1
		EFd 5%	35.3	30.0	60.0	25.5	44.8	33.3	84.0
		BR20	0.37	0.31	0.58	0.27	0.44	0.33	0.82
	BR-NiB	AUC	***0.94±0.01***	***0.91±0.04***	0.89±0.06	0.69±0.04	***0.91±0.02***	***0.91±0.03***	***0.99±0.01***
		EFd 1%	***82.8***	***90.0***	***60.0***	***45.1***	***49.3***	***35.9***	***92.6***
		EFd 5%	***89.1***	***90***	***80.0***	***51.0***	***71.6***	***71.8***	***97.5***
		BR20	***0.90***	***0.92***	***0.76***	***0.54***	***0.71***	***0.68***	***0.96***

The best values are shown in bold and italics. Only those AUC values that are outside the error margin are highlighted. The Wilcoxon statistic[17] was used for the AUC error estimation. The DUD (A Database of Useful (Docking) Decoys) benchmarking sets: COX2 (348 ligs, 12,462 decs), RXRα (20 ligs, 706 decs), MR (15 ligs, 535 decs), PDE5 (51 ligs, 1,808 decs), ER_{ag} (67 ligs, 2,352 decs), ER_{antag} (39 ligs, 1,394 decs), and PPARγ (81 ligs, 2,906 decs).

[(1)] Training/test set ratios (100:100): the percentage of ligands used in the training (100 %) in relation to the percentage used in the testing (100 %).

[(2)] Methods: flexible docking (PLANTS) and brute force negative image-based optimization (BR-NiB) with the equal shape/ESP (0.5/0.5) weight.

Table S6. The training enrichment for docking and brute force negative image-based optimization.

Train/ test(1)	Method(2)	Yield	COX2	RXRα	MR	NEU	PDE5	ER	PPARγ
70:30	Docking	AUC	0.65±0.03	0.76±0.04	0.58±0.06	0.84±0.05	0.79±0.02	0.77±0.03	0.84±0.02
		EFd 1%	5.2	10.0	6.9	3.3	12.5	27.4	24.6
		EFd 5%	20.0	35.0	27.6	33.3	30.8	40.2	55.9
		BR20	0.21	0.33	0.24	0.28	0.30	0.41	0.49
	BR-NiB	AUC	***0.79±0.02***	***0.97±0.01***	0.68±0.04	***0.96±0.02***	0.81±0.02	0.81±0.02	0.83±0.02
		EFd 1%	***37.8***	***76.9***	***27.7***	***80.9***	***24.1***	***40.7***	23.1
		EFd 5%	***50.2***	***92.3***	***47.7***	***86.8***	***45.3***	***56.8***	52.1
		BR20	***0.52***	***0.87***	***0.46***	***0.87***	***0.40***	***0.57***	0.46
	BR-NiB + shape only	AUC	***0.83±0.01***	***0.84±0.03***	**0.73±0.04**	***0.97±0.01***	***0.83±0.01***	0.64±0.02	0.85±0.01
		EFd 1%	***41.0***	***44.0***	***24.6***	***76.5***	***27.0***	***33.3***	***41.4***
		EFd 5%	***59.9***	***68.1***	***40.0***	***89.7***	***49.6***	***41.8***	***57.4***
		BR20	***0.58***	***0.64***	***0.39***	***0.86***	***0.46***	***0.42***	***0.57***
	Ligand-based rescoring	AUC	0.70±0.02	0.25±0.02	0.58±0.04	0.52±0.04	0.61±0.02	0.58±0.02	0.33±0.01
		EFd 1%	19.2	1.1	10.8	2.9	9.4	5.9	2.1
		EFd 5%	28.3	3.3	23.1	13.2	20.1	12.5	6.5
		BR20	0.30	0.03	0.23	0.11	0.20	0.13	0.06
10:90	Docking	AUC	0.68±0.05	0.74±0.08	0.47±0.10	0.82±0.09	0.83±0.04	0.78±0.04	0.83±0.04
		EFd 1%	9.3	0.0	0.0	0.0	15.4	13.2	27.1
		EFd 5%	18.6	15.4	22.2	44.4	25.6	28.9	54.2
		BR20	0.22	0.15	0.15	0.36	0.29	0.33	0.50
	BR-NiB	AUC	0.76±0.04	***0.93±0.05***	***0.84±0.08***	***1.00±0.01***	0.81±0.04	0.72±0.05	0.81±0.04
		EFd 1%	***32.8***	***92.3***	***66.7***	***100.0***	***28.2***	***55.3***	***39.6***
		EFd 5%	***47.5***	***92.3***	***66.7***	***100.0***	***53.8***	***55.3***	***58.3***
		BR20	***0.49***	***0.91***	***0.66***	***0.98***	***0.50***	***0.59***	***0.54***
	BR-NiB + shape only	AUC	***0.80±0.03***	0.79±0.08	***0.78±0.09***	***1.00±0.01***	0.86±0.04	***0.83±0.04***	0.82±0.04
		EFd 1%	***42.6***	***69.2***	***44.4***	***88.9***	***46.2***	***55.3***	***39.6***
		EFd 5%	***55.7***	***69.2***	***66.6***	***100.0***	***66.7***	***68.4***	***58.3***
		BR20	***0.58***	***0.73***	***0.63***	***0.96***	***0.60***	***0.67***	***0.57***
	Ligand-based rescoring	AUC	0.65±0.04	0.16±0.04	0.63±0.10	0.64±0.10	0.56±0.05	0.56±0.05	0.30±0.03
		EFd 1%	11.5	0.0	11.1	0.0	10.3	13.2	0.0
		EFd 5%	21.3	0.0	33.3	11.1	15.4	15.8	2.1
		BR20	0.22	0.00	0.28	0.14	0.17	0.18	0.02

The best values are underscored, and rescoring values are shown in bold and italics, if improved in comparison to the docking or ligand-based rescoring of each set (70 %, 30 %, 10 % or 90 %). Only those AUC values that are outside the error margin are highlighted. The Wilcoxon statistic[15] was used for the AUC error estimation.

(1) Train/test set ratios (100:0, 70:30, 10:90): the percentage of ligands used in the negative image-based (NIB) model training (100 %, 70 %, 10 %) in relation to the percentage applied in the testing (100 %, 30 %, 90 %).

(2) Methods: flexible molecular docking using PLANTS and brute force negative image-based optimization (BR-NiB; Figure 2) either with equal shape/electrostatic potential (0.5/0.5) weight or using only the shape (1.0/0.0). In the ligand-based rescoring, the docking poses are directly compared without geometry optimization in SHAEP to the ligand 3D conformers co-crystallized with the target proteins (Table S1).

Table S7 Docking and brute force negative image-based optimization performance for 70 % training set with five extra DUD-E targets and alternative PPARγ structure (PDB: 3GBK).

**Method** [(2)]	**Yield**	**AKT1**	**DRD3**	**ACES**	**COMT**	**FAK1**	**PPARγ** [(3)]
Docking	AUC	0.63±0.02	0.63±0.02	0.37±0.01	0.64±0.06	0.79±0.03	0.83±0.01
	EFd 1%	2.4	0.9	1.3	0.0	21.4	22.5
	EFd 5%	28.3	6.3	4.1	10.7	34.3	47.4
	BR20	0.24	0.08	0.04	0.13	0.36	0.43
BR-NiB	AUC	***0.73±0.02***	***0.79±0.02***	***0.69±0.02***	***0.85±0.05***	***0.93±0.02***	***0.87±0.01*** (0.83±0.01)
	EFd 1%	***22.4***	***22.6***	***20.5***	***28.6***	***74.3***	***53.6*** (43.5)
	EFd 5%	***42.9***	***56.8***	***29.0***	***64.3***	***85.7***	***71.3*** (63.6)
	BR20	***0.40***	***0.51***	***0.33***	***0.53***	***0.83***	***0.67*** (0.60)

The best values are shown in bold and italics. Only those AUC values that are outside the error margin are highlighted. The Wilcoxon statistic[17] was used for the AUC error estimation.

(1) Training/test set ratio (70:30): the percentage of ligands used in the training (70 %) in relation to the percentage used in the testing (30 %).

(2) Methods: flexible docking (PLANTS) and brute force negative image-based optimization (BR-NiB) with the equal shape/ESP (0.5/0.5) weight.

(3) The shape only (1.0/0.0 of shape/ESP) weight of scoring results are shown in parentheses.

Table S8. Testing of brute force negative image-based optimized models trained with 70 % and 10 % sets against the 100 % sets.

Train/ test	Method [2]	Yield	COX2	RXRα	MR	NEU	PDE5	ER	PPARγ
70: 100	BR-NiB	AUC	***0.78±0.01***	***0.97±0.01***	***0.71±0.03***	***0.95±0.02***	***0.81±0.01***	***0.82±0.01***	***0.83±0.01***
		EFd 1%	***37.2***	***78.6***	27.7	74.5	***22.1***	39.9	24.6
		EFd 5%	49.4	***91.6***	46.8	83.7	***42.2***	***56.1***	51.2
		BR20	***0.51***	***0.87***	0.45	0.83	0.38	***0.55***	0.46
	BR-NiB + shape only	AUC	***0.83±0.01***	***0.85±0.02***	***0.73±0.03***	***0.96±0.01***	***0.85±0.01***	***0.66±0.02***	***0.84±0.01***
		EFd 1%	***39.8***	45.8	28.7	75.5	24.9	33.2	***40.5***
		EFd 5%	***58.6***	69.5	***41.5***	***87.8***	47.5	43.1	56.4
		BR20	**0.57**	0.65	0.41	***0.85***	0.44	0.43	0.56
10: 100	BR-NiB	AUC	***0.77±0.01***	***0.95±0.01***	***0.77±0.03***	***0.94±0.02***	0.76±0.01	0.66±0.02	***0.81±0.01***
		EFd 1%	29.9	74.0	9.6	60.2	6.8	35.8	14.0
		EFd 5%	48.0	85.5	35.1	77.6	22.9	45.7	44.2
		BR20	0.46	0.81	0.31	0.73	0.22	0.46	0.39
	BR-NiB + shape only	AUC	***0.82±0.01***	0.69±0.03	***0.73±0.03***	***0.93±0.02***	0.79±0.01	***0.76±0.01***	0.81±0.01
		EFd 1%	***40.0***	26.7	19.1	53.1	16.6	33.2	25.6
		EFd 5%	57.2	37.4	38.3	72.4	39.9	***47.5***	43.6
		BR20	0.55	0.39	0.37	0.69	0.36	***0.48***	0.44

The rescoring values are shown in bold and italics, if equal or improved in comparison to the BR-NiB of the original 100:0 training/test set division (Table 1). Only those AUC values that are outside the error margin are highlighted. The Wilcoxon statistic[15] was used for the AUC error estimation.

(1) Train/test set ratios (70:100, 10:100): the percentage of ligands used in the training (70 %, 10 %) in relation to the percentage used in the testing (100 %).

(2) Methods: flexible docking (PLANTS) and brute force negative image-based optimization (BR-NiB) either with the equal shape/ESP (0.5/0.5) weight or the shape only (1.0/0.0).

Table S9. The test set enrichment for the ligand-based rescoring.

Train/ test	Yield	COX2	RXRα	MR	NEU	PDE5	ER	PPARγ
100:100	AUC	***0.69±0.01***	0.26±0.02	***0.61±0.03***	0.50±0.03	0.61±0.02	0.56±0.02	0.32±0.01
	EFd 1%	***20.0***	2.3	***11.7***	3.1	9.3	5.2	2.5
	EFd 5%	***28.3***	4.6	***25.5***	11.2	20.6	11.2	6.8
	BR20	***0.30***	0.04	***0.25***	0.10	0.20	0.12	0.07
70:30	AUC	***0.67±0.03***	0.26±0.03	***0.66±0.06***	0.46±0.05	0.61±0.03	0.52±0.03	0.32±0.02
	EFd 1%	***20.7***	0.0	***10.3***	***3.3***	7.5	4.3	3.4
	EFd 5%	***28.1***	7.5	***34.5***	6.7	21.7	9.4	7.5
	BR20	***0.30***	0.06	***0.30***	0.07	0.19	0.09	0.07
10:90	AUC	***0.69±0.01***	0.27±0.02	***0.60±0.03***	0.48±0.03	0.61±0.02	0.56±0.02	0.32±0.01
	EFd 1%	***20.4***	1.7	***10.6***	***3.4***	9.2	4.3	2.8
	EFd 5%	***28.8***	5.1	***23.5***	11.2	20.9	11.2	7.3
	BR20	***0.31***	0.04	***0.25***	0.10	0.20	0.12	0.07

The values that are as good as or better than the best docking results (Table 1) are shown in bold and italics. Underlined, if better than R-NiB method (Table S2).

In the ligand-based rescoring (PLANTS + X-ray/SHAEP), the docking poses are directly compared without geometry optimization in SHAEP to the ligand 3D conformers co-crystallized with the target proteins (Table S1). The testing was performed using equal shape/electrostatic potential (0.5/0.5) weight. The Wilcoxon statistic[15] was used for the AUC error estimation.

Table S10. The composition of negative images before and after brute force image-based optimization.

Target protein	Method[(1)]	Train/ test [(2)]	No. of Gens	No. of cavity atoms[(3)]	No. of positive cavity atoms[(4)]	No. of negative cavity atoms[(5)]	No of neutral cavity atoms[(6)]
COX2	BR-NiB	100:0	9	35/44	2/2	2/2	31/40
		70:30	13	31/44	2/2	2/2	27/40
		10:90	6	38/44	2/2	2/2	34/40
	BR-NiB + shape only	100:0	15[(*)]	29/44	2/2	2/2	25/40
		70:30	15[(*)]	29/44	2/2	2/2	25/40
		10:90	14	30/44	2/2	2/2	26/40
RXRα	BR-NiB	100:0	23	33/56	0/0	2/2	31/53
		70:30	21	35/56	0/0	2/2	33/53
		10:90	19	37/56	0/0	2/2	35/53
	BR-NiB + shape only	100:0	22	34/56	0/0	2/2	32/53
		70:30	25	31/56	0/0	2/2	29/53
		10:90	26	30/56	0/0	2/2	28/53
MR	BR-NiB	100:0	17	40/57	1/1	1/3	38/53
		70:30	15	42/57	1/1	1/3	40/53
		10:90	19	38/57	1/1	3/3	34/53
	BR-NiB + shape only	100:0	16	41/57	0/1	1/3	40/53
		70:30	15	42/57	0/1	1/3	41/53
		10:90	18	39/57	0/1	2/3	37/53
NEU	BR-NiB	100:0	34	45/79	4/5	5/6	36/68
		70:30	27	52/79	4/5	5/6	43/68
		10:90	24	55/79	5/5	6/6	44/68
	BR-NiB + shape only	100:0	30[(*)]	49/79	4/5	6/6	39/68
		70:30	30[(*)]	49/79	4/5	6/6	39/68
		10:90	31	48/79	3/5	5/6	40/68
PDE5	BR-NiB	100:0	74	55/129	2/4	3/3	50/122
		70:30	77	52/129	3/4	3/3	46/122
		10:90	46	83/129	3/4	3/3	77/122
	BR-NiB + shape only	100:0	72	57/129	2/4	1/3	54/122
		70:30	71	58/129	2/4	2/3	54/122
		10:90	77	52/129	1/4	1/3	50/122
ER	BR-NiB	100:0	41	38/79	2/3	2/2	34/74
		70:30	40	39/79	2/3	2/2	35/74
		10:90	30	49/79	2/3	1/2	46/74
	BR-NiB + shape only	100:0	27	52/79	3/3	2/2	47/74
		70:30	21	58/79	3/3	2/2	53/74
		10:90	35	44/79	3/3	2/2	39/74
PPARγ	BR-NiB	100:0	77	67/144	5/5	0/3	62/136
		70:30	79	65/144	5/5	0/3	60/136
		10:90	82	62/144	5/5	0/3	57/136
	BR-NiB + shape only	100:0	74	70/144	0/5	2/3	68/136
		70:30	69	75/144	1/5	2/3	72/136
		10:90	68	76/144	2/5	1/3	73/136

(1) Screening methods: brute force negative image-based optimization (BR-NiB; Figure 2) either with equal shape/electrostatic potential (0.5/0.5) weight or using only the shape (1.0/0.0).
(2) Train/test set ratios (100:100, 70:30, 10:90): the percentage of ligands used in the model training (100 %, 70 %, 10 %) in relation to the percentage applied in the testing (100 %, 30 %, 90 %). The test set percentage is underlined in the Ratio column.
Final optimized model/input generation #0 model: (3) number (No.) of cavity points in total, (4) positively charged cavity atoms, (5) negatively charged cavity atoms and (6) neutral filler atoms or cavity atoms.
(*) The BR-NiB optimized NIB models, optimized using 100 % and 70 % training sets, are of identical size but their compositions differ by two cavity atoms.

Table S11. Comparing ligands to the negative image-based models before and after brute force image-based optimization.

Target protein	Method (1)	Train/ test(2)	Input model (Gen #0)		Optimized model		DUD-E active (3)	
			V (Å^3)	MW (g/mol)	V (Å^3)	MW (g/mol)	V (Å^3)	MW (g/mol)
COX2	BR-NiB	100:0<br>70:30<br>10:90	448	544	388<br>366<br>404	436<br>388<br>472	284	375
	BR-NiB + shape only	100:0<br>70:30<br>10:90	448	544	363<br>358<br>365	364<br>364<br>376	284	375
RXRα	BR-NiB	100:0<br>70:30<br>10:90	548	699	402<br>397<br>419	408<br>432<br>456	333	408
	BR-NiB + shape only	100:0<br>70:30<br>10:90	548	699	412<br>411<br>370	398<br>422<br>372	333	408
MR	BR-NiB	100:0<br>70:30<br>10:90	493	705	408<br>441<br>397	488<br>512<br>476	318	410
	BR-NiB + shape only	100:0<br>70:30<br>10:90	493	705	411<br>417<br>409	498<br>510<br>480	318	410
NEU	BR-NiB	100:0<br>70:30<br>10:90	661	995	502<br>555<br>603	579<br>663<br>707	264	336
	BR-NiB + shape only	100:0<br>70:30<br>10:90	661	995	569<br>565<br>537	633<br>633<br>613	264	336
PDE5	BR-NiB	100:0<br>70:30<br>10:90	926	1575	583<br>565<br>776	685<br>649<br>1021	347	447
	BR-NiB + shape only	100:0<br>70:30<br>10:90	926	1575	648<br>647<br>600	695<br>713<br>633	347	447
ER	BR-NiB	100:0<br>70:30<br>10:90	684	967	449<br>443<br>549	472<br>484<br>599	327	414
	BR-NiB + shape only	100:0<br>70:30<br>10:90	684	967	570<br>601<br>525	643<br>715<br>546	327	414
PPARγ	BR-NiB	100:0<br>70:30<br>10:90	1228	1758	825<br>786<br>729	815<br>791<br>755	365	464
	BR-NiB + shape only	100:0<br>70:30<br>10:90	1228	1758	835<br>876<br>824	853<br>915<br>923	365	464

(1) Screening methods: brute force negative image-based optimization (BR-NiB; Figure 2) either with equal shape/ESP (0.5/0.5) weight or using only the shape (1.0/0.0).
(2) Train/test set ratios (100:100, 70:30, 10:90): the percentage of ligands used in the negative image-based (NIB) model training (100 %, 70 %, 10 %) in relation to the percentage applied in the testing (100 %, 30 %, 90 %).
(3) Molecular weight (MW) and molecule volumes (V) are given as average values.
The NIB models were converted to MAE format using MOL2CONVERT in MAESTRO 2018-1 (Schrödinger, LLC, New York, NY, USA, 2018). V and MW were calculated using volume_calc.py and calculate_properties.py in MAESTRO, respectively.

Table S12. Fitting negative image to the shape/ESP profile of the active ligands.

**Train /test** (1)	**Method** (2)	**Yield**	**COX2**	**RXRα**	**MR**	**NEU**	**PDE5**	**ER**	**PPARγ**
100:100	Shape/ ESP fitting	AUC	0.74±0.01	***0.95±0.01***	0.63±0.03	0.88±0.02	0.54±0.01	0.57±0.02	***0.83±0.01***
		EFd 1%	12.4	64.1	11.7	43.9	0.75	4.7	13.8
		EFd 5%	36.3	85.5	27.7	59.2	6.5	13.1	38.4
		BR20	0.33	0.80	0.27	0.58	0.08	0.13	0.35
	Shape/ ESP fitting + BR-NiB	AUC	0.77±0.01	***0.96±0.01***	**0.66±0.03**	0.92±0.02	0.64±0.02	0.59±0.02	***0.85±0.01***
		EFd 1%	22.8	77.1	19.1	61.2	9.5	11.7	***27.1***
		EFd 5%	43.4	88.5	40.4	80.6	20.1	24.0	47.5
		BR20	0.42	0.85	0.39	0.76	0.19	0.23	0.46
100:100	Shape fitting	AUC	0.81±0.01	***0.95±0.01***	**0.67±0.03**	0.92±0.02	0.64±0.02	***0.66±0.02***	0.77±0.01
		EFd 1%	25.5	***66.4***	9.6	54.1	3.3	6.0	8.1
		EFd 5%	46.2	***83.2***	29.8	65.3	15.3	16.4	26.0
		BR20	0.44	***0.80***	0.28	0.66	0.15	0.17	0.26
	Shape fitting + BR-NiB	AUC	0.81±0.01	***0.96±0.01***	**0.69±0.03**	0.92±0.02	0.69±0.01	***0.71±0.02***	0.79±0.01
		EFd 1%	26.4	***77.9***	26.6	56.1	9.8	15.4	14.3
		EFd 5%	50.8	***87.8***	36.2	72.4	26.4	28.7	33.5
		BR20	0.47	***0.84***	0.37	0.70	0.24	0.28	0.32

The rescoring values are shown in bold and italics, if equal or improved in comparison to the BR-NiB of the original 100:0 training/test set division (Table 1). Only those AUC values that are outside the error margin are highlighted. The Wilcoxon statistic[15] was used for the AUC error estimation.

(1) Train/test set ratios (100:100) indicates that all ligands were used in the model training and testing.

(2) Methods: the NIB model was fitted to the docked active ligands using the shape only or equal shape/ESP score in SHAEP. Accordingly, the cavity atom composition, which generated the best average score for the top-ranked docking poses, was tested directly in negative image-based rescoring (R-NiB). Furthermore, the shape or shape/ESP fitted NIB models were processed also using brute force negative image-based optimization (BR-NiB) either with the equal shape/ESP (0.5/0.5) weight or the shape only (1.0/0.0).

Table S13. The testing set enrichment of brute force negative image-based rescoring with alternative docking software.

**Software / Method**	**Yield**	**Train/test 100:100** (1)				**Train/test 70:30** (1)				**Train/test 10:90** (1)			
		**COX2**	**RXRα**	**NEU**	**MR**	**COX2**	**RXRα**	**NEU**	**MR**	**COX2**	**RXRα**	**NEU**	**MR**
GLIDE SP Docking	AUC	0.74±0.01	0.83±0.02	0.82±0.03	N/A (2)	0.76±0.03	0.78±0.04	0.82±0.05	N/A (2)	0.76±0.01	0.80±0.02	0.82±0.03	N/A (2)
	EFd 1%	37.2	65.6	18.4	N/A (2)	36.7	67.5	0.0	N/A (2)	38.2	63.6	20.2	N/A (2)
	EFd 5%	46.7	77.1	43.9	N/A (2)	47.7	72.5	50.0	N/A (2)	47.9	76.3	43.8	N/A (2)
	BR20	0.48	0.76	0.41	N/A (2)	0.49	0.74	0.42	N/A (2)	0.49	0.74	0.41	N/A (2)
GLIDE SP + BR-NiB	AUC	0.74±0.01	0.83±0.02	***0.97±0.01***	N/A (2)	0.70±0.03	0.82±0.04	***0.96±0.03***	N/A (2)	0.74±0.01	0.82±0.02	***0.95±0.02***	N/A (2)
	EFd 1%	***40.7***	***68.7***	***91.8***	N/A (2)	35.2	65.0	***76.7***	N/A (2)	38.2	61.0	***68.5***	N/A (2)
	EFd 5%	***51.7***	***79.4***	***94.9***	N/A (2)	***49.2***	***75.0***	***86.7***	N/A (2)	***51.3***	71.2	***86.5***	N/A (2)
	BR20	***0.53***	***0.78***	***0.93***	N/A (2)	0.47	***0.76***	***0.86***	N/A (2)	***0.51***	0.72	***0.81***	N/A (2)
DOCK Docking	AUC	0.64±0.01	0.52±0.03	0.82±0.03	0.42±0.04	0.63±0.03	0.54±0.05	0.81±0.05	0.43±0.05	0.65±0.02	0.50±0.03	0.82±0.03	N/A (2)
	EFd 1%	11.3	8.4	14.3	0.0	9.4	10.0	10.0	0.0	11.5	8.5	13.5	N/A (2)
	EFd 5%	22.1	13.0	36.7	0.0	18.8	15.0	30.0	0.0	23.0	12.7	37.1	N/A (2)
	BR20	0.21	0.13	0.35	0.02	0.18	0.16	0.30	0.01	0.22	0.13	0.35	N/A (2)
DOCK + BR-NiB	AUC	***0.73±0.01***	***0.96±0.01***	***0.96±0.01***	***0.59±0.03***	***0.71±0.03***	***0.78±0.04***	***0.94±0.03***	***0.57±0.06***	***0.72±0.02***	***0.74±0.03***	***0.90±0.02***	N/A (2)
	EFd 1%	***27.1***	***74.0***	***74.5***	***21.3***	***17.2***	***57.5***	***60.0***	***17.2***	***24.6***	***50.8***	***48.3***	N/A (2)
	EFd 5%	***46.4***	***88.5***	***89.8***	***29.8***	***36.7***	***72.5***	***76.7***	***27.6***	***42.0***	***57.6***	***64.0***	N/A (2)
	BR20	***0.44***	***0.85***	***0.85***	***0.47***	***0.37***	***0.70***	***0.73***	***0.29***	***0.40***	***0.60***	***0.62***	N/A (2)
GOLD Docking	AUC	0.71±0.01	0.76±0.02	0.69±0.03	0.47±0.01	0.71±0.03	0.75±0.05	0.70±0.05	0.48±0.05	0.70±0.01	0.76±0.03	0.69±0.03	0.48±0.03
	EFd 1%	12.8	16.8	2.0	2.1	8.1	12.5	3.3	0.0	12.2	16.9	2.2	2.4
	EFd 5%	38.1	35.1	9.2	7.4	37.0	35.0	13.3	6.9	37.0	36.4	10.1	8.3
	BR20	0.34	0.35	0.13	0.08	0.33	0.35	0.17	0.08	0.33	0.37	0.13	0.08
GOLD + BR-NiB	AUC	***0.78±0.01***	***0.94±0.01***	***0.96±0.01***	***0.71±0.03***	***0.78±0.02***	***0.93±0.03***	***0.97±0.02***	***0.78±0.05***	***0.78±0.01***	***0.92±0.02***	***0.94±0.02***	***0.65±0.03***
	EFd 1%	***37.7***	***76.3***	***81.6***	***36.2***	***30.2***	***77.5***	***76.7***	***27.6***	***35.0***	***70.3***	***59.6***	***16.5***
	EFd 5%	***53.6***	***85.5***	***89.8***	***47.9***	***48.1***	***85.0***	***86.7***	***55.2***	***53.2***	***78.0***	***80.9***	***34.1***
	BR20	***0.52***	***0.84***	***0.88***	***0.47***	***0.48***	***0.84***	***0.85***	***0.49***	***0.50***	***0.77***	***0.75***	***0.32***

Brute force negative image-based (BR-NiB; Figure 2) results that are higher than the original docking values are shown in bold and italics. The rescoring results that are as good as or better than the equivalent BR-NiB results for PLANTS (Table 1) are underlined. The equal shape/electrostatics potential (50/50) weight was applied in the similarity scoring. The Wilcoxon statistic[15] was used for the AUC error estimation.

(1) Train/test set ratios (100:100, 70:30, 10:90): the percentage of ligands used in the model training (100 %, 70 %, 10 %) in relation to the percentage applied in the testing (100 %, 30 %, 90 %).

(2) Not available (N/A): The results could not be calculated because the docking software skipped over 50 % of active ligands included in the training and/or test set.

Table S14. The training set enrichment of brute force negative image-based rescoring with alternative docking software.

**Software / Method**	**Yield**	**Train/test 70:30 (1)**				**Train/test 10:90 (1)**			
		**COX2**	**RXRα**	**NEU**	**MR**	**COX2**	**RXRα**	**NEU**	**MR**
GLIDE SP Docking	AUC	0.74±0.02	0.82±0.03	0.81±0.03	N/A (2)	0.70±0.04	0.90±0.06	0.78±0.09	N/A (2)
	EFd 1%	37.5	63.7	22.1	N/A (2)	36.1	69.2	0.0	N/A (2)
	EFd 5%	46.6	79.1	41.2	N/A (2)	44.3	84.6	44.4	N/A (2)
	BR20	0.48	0.76	0.40	N/A (2)	0.46	0.85	0.38	N/A (2)
GLIDE SP + BR-NiB	AUC	0.75±0.02	0.84±0.03	***0.97±0.01***	N/A (2)	0.70±0.04	0.89±0.06	***1.00±0.00***	N/A (2)
	EFd 1%	***43.0***	***69.2***	***94.1***	N/A (2)	***39.3***	***84.6***	***100.0***	N/A (2)
	EFd 5%	***53.1***	79.1	***97.1***	N/A (2)	***52.5***	84.6	***100.0***	N/A (2)
	BR20	***0.55***	***0.78***	***0.97***	N/A (2)	***0.53***	***0.87***	***0.99***	N/A (2)
DOCK Docking	AUC	0.65±0.02	0.50±0.04	0.83±0.03	0.51±0.04	0.59±0.03	0.63±0.08	0.83±0.08	N/A (2)
	EFd 1%	11.7	7.7	17.6	0.0	9.8	7.7	22.2	N/A (2)
	EFd 5%	23.5	12.1	39.7	1.5	19.7	15.4	33.3	N/A (2)
	BR20	0.23	0.12	0.37	0.03	0.20	0.18	0.32	N/A (2)
DOCK + BR-NiB	AUC	***0.74±0.02***	***0.74±0.03***	***0.62±0.03***	***0.63±0.04***	0.68±0.04	***0.81±0.07***	***1.00±0.01***	N/A (2)
	EFd 1%	***30.0***	***57.1***	***40.7***	***23.1***	***29.5***	***76.9***	***100.0***	N/A (2)
	EFd 5%	***48.9***	***63.7***	***46.7***	***36.9***	***41.0***	***76.9***	***100.0***	N/A (2)
	BR20	***0.47***	***0.65***	***0.48***	***0.36***	***0.42***	***0.79***	***0.98***	N/A (2)
GOLD Docking	AUC	0.70±0.02	0.76±0.03	0.68±0.03	0.45±0.03	0.67±0.04	0.75±0.08	0.71±0.10	0.39±0.09
	EFd 1%	15.6	18.7	0.0	1.5	23.0	15.4	0.0	0.0
	EFd 5%	38.1	35.2	8.8	6.1	36.1	23.1	0.0	0.0
	BR20	0.35	0.36	0.11	0.06	0.39	0.25	0.12	0.04
GOLD + BR-NiB	AUC	***0.78±0.02***	***0.95±0.02***	***0.96±0.02***	***0.69±0.04***	***0.77±0.04***	***0.92±0.05***	***0.95±0.05***	***0.75±0.09***
	EFd 1%	***39.1***	***75.8***	***80.9***	***30.8***	***36.1***	***84.6***	***77.8***	***55.6***
	EFd 5%	***53.7***	***84.6***	***91.2***	***41.5***	***50.8***	***92.3***	***88.9***	***66.7***
	BR20	***0.53***	***0.83***	***0.88***	***0.41***	***0.51***	***0.92***	***0.87***	***0.65***

Brute force negative image-based (BR-NiB; Figure 2) results that are higher than the original docking values are shown in bold and italics. The rescoring results that are as good as or better than the equivalent BR-NiB results for PLANTS (Table S8) are underlined. The equal shape/electrostatics potential (50/50) weight was applied in the similarity scoring. The Wilcoxon statistic[15] was used for the AUC error estimation.

(1) Train/test set ratios (70:30, 10:90): the percentage of ligands used in the model training (70 %, 10 %) in relation to the percentage applied in the testing (30 %, 90 %).

(2) Not available (N/A): The results could not be calculated because the docking software skipped over 50 % of active ligands included in the training and/or test set.

Table S15. The cross-use of brute force optimized models between docking software.

Docking	Model (1)	Yield	COX2	RXRα	NEU	MR
PLANTS	GLIDE SP + BR-NiB	AUC	***0.78±0.01***	***0.96±0.01***	***0.95±0.01***	***0.77±0.03***
		EFd 1%	***29.4***	***72.5***	***73.5***	***11.7***
		EFd 5%	***51.3***	***89.3***	***81.6***	***30.9***
		BR20	***0.49***	***0.83***	***0.81***	***0.29***
	DOCK + BR-NiB	AUC	***0.78±0.01***	***0.96±0.01***	***0.96±0.01***	***0.65±0.03***
		EFd 1%	***25.5***	***70.2***	***70.4***	***5.3***
		EFd 5%	***51.5***	***88.5***	***82.7***	***23.4***
		BR20	***0.47***	***0.83***	***0.79***	***0.24***
	GOLD + BR-NiB	AUC	***0.78±0.01***	***0.96±0.01***	***0.95±0.02***	***0.72±0.03***
		EFd 1%	***34.3***	***75.6***	***60.2***	***24.4***
		EFd 5%	***53.1***	***88.5***	***80.6***	***40.4***
		BR20	***0.51***	***0.84***	***0.76***	***0.41***
GOLD	GLIDE SP + BR-NiB	AUC	***0.77±0.01***	***0.94±0.01***	***0.94±0.02***	***0.74±0.03***
		EFd 1%	***29.4***	***76.3***	***77.6***	***14.9***
		EFd 5%	***50.6***	***85.5***	***83.7***	***29.8***
		BR20	***0.48***	***0.84***	***0.84***	***0.30***
	DOCK + BR-NiB	AUC	***0.78±0.01***	***0.92±0.02***	***0.96±0.01***	***0.62±0.03***
		EFd 1%	***29.0***	***69.5***	***75.5***	***9.6***
		EFd 5%	***49.4***	***80.2***	***86.7***	***26.6***
		BR20	***0.47***	***0.79***	***0.85***	***0.25***
	PLANTS + BR-NiB	AUC	***0.77±0.01***	***0.93±0.02***	***0.95±0.02***	***0.69±0.03***
		EFd 1%	***32.9***	***69.5***	***78.6***	***22.3***
		EFd 5%	***47.8***	***82.4***	***87.8***	***39.4***
		BR20	***0.47***	***0.80***	***0.86***	***0.38***
GLIDE SP	PLANTS + BR-NiB	AUC	0.73±0.01	0.83±0.02	***0.97±0.01***	N/A (2)
		EFd 1%	***37.5***	***67.2***	***87.8***	N/A (2)
		EFd 5%	***49.7***	72.5	***93.9***	N/A (2)
		BR20	***0.49***	0.73	***0.90***	N/A (2)
	DOCK + BR-NiB	AUC	0.73±0.01	0.83±0.02	***0.97±0.01***	N/A (2)
		EFd 1%	***39.8***	***66.4***	***87.8***	N/A (2)
		EFd 5%	***51.0***	74.8	***93.9***	N/A (2)
		BR20	***0.51***	0.75	***0.92***	N/A (2)
	GOLD + BR-NiB	AUC	0.74±0.01	0.83±0.02	***0.96±0.01***	N/A (2)
		EFd 1%	***40.0***	***67.2***	***81.6***	N/A (2)
		EFd 5%	***50.6***	74.1	***92.9***	N/A (2)
		BR20	***0.52***	0.75	***0.89***	N/A (2)
DOCK	GLIDE SP + BR-NiB	AUC	***0.73±0.01***	***0.75±0.02***	***0.92±0.02***	***0.55±0.03***
		EFd 1%	***26.2***	***54.2***	***65.3***	0.0
		EFd 5%	***45.1***	***64.1***	***79.6***	***11.7***
		BR20	***0.43***	***0.64***	***0.78***	***0.13***
	PLANTS + BR-NiB	AUC	***0.72±0.01***	***0.74±0.03***	***0.95±0.02***	***0.57±0.03***
		EFd 1%	***22.5***	***51.1***	***71.4***	***5.3***
		EFd 5%	***40.7***	***60.3***	***79.6***	***16.0***
		BR20	***0.40***	***0.62***	***0.79***	***0.18***
	GOLD + BR-NiB	AUC	***0.72±0.01***	***0.75±0.02***	***0.94±0.02***	***0.56±0.03***
		EFd 1%	***25.3***	***53.4***	***65.3***	***7.4***
		EFd 5%	***43.2***	***61.8***	***80.6***	***14.9***
		BR20	***0.42***	***0.63***	***0.77***	***0.17***

Brute force negative image-based optimization (BR-NiB; Figure 2) results that are higher than the original docking values are shown in bold and italics. The rescoring results that are as good as or better than the equivalent BR-NiB results for PLANTS (Table 2) are underlined. All ligands were used in the training/testing (100:100) and the equal shape/electrostatics potential (50/50) weight was applied in the similarity scoring. The Wilcoxon statistic[15] was used for the AUC error estimation.

(1) The BR-NiB models were created based on the docking solutions of GLIDE SP, GOLD or DOCK.

(2) Not available (N/A): The docking software skipped over 50 % of active ligands in the training and/or test set.

Table S16. The computing demands of brute force negative image-based optimization.

	COX2	MR	NEU
**No. of cavity atoms at Gen #0**	44	57	79
**No. of cavity atoms at Gen #X**	30	41	51
**No. of compounds**	176,830	39,090	48,860
**No. of subsets**	18	4	5
**Max no. of compounds / subset**	10,000	10,000	10,000
**RAM / CPU**	~400 MB	~390 MB	~330 MB
**Gen #0 with 15 CPUs**	21 min	8 min	12 min
**Gen #X with 15 CPUs**	17 min	6 min	8 min
**No. of gens**	13	15	27
**Total duration with 15 CPUs**	5 h 47 min	2 h	4 h 43 min

The compound sets were divided into subsets containing 10,000 molecules to reduce memory consumption and to assist with the parallel computing. Gen #X corresponds the generation in which the improvement halts.
Train/test set ratio (70/30): the percentage of active/inactive ligands used in the model training (70 %) in relation to the percentage applied in the testing (30 %).
The computing performance was benchmarked with LINUX desktop computer using Intel Xeon CPUs (E5-2630 v4, 2.20 GHz) and RAM 16 GB.

Table S17. One generation optimization of negative image-based models.

		COX2	RXRα	MR	NEU	PDE5	ER	PPARγ
**Yield at Gen #0**	AUC	0.72±0.01	0.86±0.02	0.66±0.03	0.83±0.03	0.58±0.02	0.62±0.02	0.78±0.01
	EFd 1%	11.5	9.2	***9.6***	3.1	0.0	2.9	13.4
	EFd 5%	30.6	34.4	21.3	29.6	1.5	4.7	36.6
	BR20	0.28	0.34	0.22	0.26	0.04	0.06	0.33
**Yield at final Gen**	AUC	***0.74±0.01***	0.94±0.01	0.64±0.03	***0.91±0.02***	0.63±0.02	***0.79±0.01***	0.83±0.01
	EFd 1%	***23.9***	***78.6***	***12.8***	***51.0***	***14.6***	***39.2***	17.8
	EFd 5%	***43.7***	***89.3***	***29.8***	***74.5***	***30.7***	***50.9***	44.2
	BR20	***0.41***	***0.85***	***0.29***	***0.68***	***0.29***	***0.52***	0.40
**No of cavity atoms**	Gen #0[(1)]	23	26	33	38	78	52	64
	Final Gen[(2)]	<u>13</u>	<u>15</u>	26	<u>31</u>	51	31	<u>47</u>

All cavity atoms lowering the enrichment at any level when removed from the input NIB (negative image-based (NIB) model) model separately (Gen #1 models) were pooled together and removed in one go. This pruned one-generation model was tested with negative image-based rescoring (R-NiB).

The enrichment values are shown in bold and italics, if improved in comparison to the docking and negative image-based rescoring (R-NiB) approaches (Table 1, Tables S2 and S3). The train/test set ratio of 100:100 was applied with 50/50 shape/electrostatic similarity score. The Wilcoxon statistic[15] was used for the AUC error estimation.

[(1)] The number of cavity atoms after removing the atoms that were not improving the yield in the first generation, *i.e.*, their removal increased the BR20 value.

[(2)] The number of cavity atoms in the final model. The final models that contained at least 20 cavity atoms less than the original BR-NiB model (Table S7) are underlined.

Table S18. The enrichment metrics of ten MUV sets.

Software / Method	Yield	S1P1	PKA	Rho-K2	HIV	HSP90	FAK1	Cath G	FXIa	FXIIa	EphA4
Docking	AUC	0.53±0.05	0.54±0.05	0.44±0.05	0.45±0.05	0.42±0.05	0.46±0.05	0.38±0.05	0.38±0.05	0.32±0.04	0.44±0.05
	EFd 1%	3.3	0.0	3.3	3.3	0.0	3.3	0.0	0.0	0.0	0.0
	EFd 5%	10.0	6.7	6.7	3.3	0.0	6.7	3.3	0.0	0.0	0.0
	BR20	0.08	0.03	0.06	0.05	0.00	0.05	0.02	0.04	0.00	0.01
BR-NiB	AUC	0.60±0.05	***0.75±0.05***	***0.71±0.05***	***0.69±0.05***	0.49±0.05	0.53±0.05	***0.65±0.05***	***0.70±0.05***	***0.69±0.05***	***0.64±0.05***
	EFd 1%	***26.7***	***13.3***	***33.3***	***23.3***	***23.3***	***23.3***	***23.3***	***26.7***	***16.7***	***13.3***
	EFd 5%	***26.7***	***46.7***	***43.3***	***40.0***	***26.7***	***23.3***	***40.0***	***43.3***	***33.3***	***43.3***
	BR20	***0.27***	***0.35***	***0.37***	***0.33***	***0.24***	***0.22***	***0.33***	***0.36***	***0.27***	***0.32***

The values are shown in bold and italics, if improved in comparison to the docking. Only those AUC values that are outside the error margin are highlighted. The testing was performed with the full data set (100:100) using equal shape/electrostatic potential (0.5/0.5) weight. The Wilcoxon statistic[15] was used for the AUC error estimation. The PDB codes for the targets structures used are the following: 3V2Y (S1P1), 4UJ1 (PKA), 7JNT (Rho-K2), 3LP1 (HIV), 6LTK (HSP90), 3BZ3 (FAK1), 1T32 (Cath G), FXIa (7MBO), FXIIa (6B77) and EphA4 (2WO2).

Table S19. Root-Mean-Square deviation: Docking and rescoring *vs.* X-ray crystallography.

Method	RMSd [(2)]	COX2	RXRα	MR	NEU	PDE5	ER	PPARγ	Total
**X-ray [(1)]**	-	8	6	5	8	4	10	18	60
**All docking poses [(3)]**	**< 1 Å**	4	4	4	3	0	1	1	17
	**< 2 Å**	7	6	5	7	2	8	4	39
	**< 3 Å**	8	6	5	8	2	8	9	46
**Docking scoring**	**< 1 Å**	2	1	4	1	0	0	0	8
	**< 2 Å**	3	3	5	3	0	4	2	20
	**< 3 Å**	3	6	5	4	0	6	4	28
**BR-NiB: Gen #0**	**< 1 Å**	2	0	4	2	0	0	0	8
	**< 2 Å**	4	3	5	5	1	1	0	19
	**< 3 Å**	4	5	5	6	1	4	1	26
**BR-NiB**	**< 1 Å**	2	3	2	2	0	1	0	10
	**< 2 Å**	2	4	2	5	0	3	1	17
	**< 3 Å**	2	6	2	6	1	6	1	24

(1) The number of docked active ligands with known poses from X-ray crystallographic studies.
(2) The Root-Mean-Square deviation (RMSd) value of the best pose suggested by the docking software, brute force negative image-based rescoring (BR-NiB) or BR-NiB: Gen #0 or negative image-based rescoring (R-NiB).
(3) All docking poses outputted by PLANTS for each active ligand (N = 10) with co-crystallized poses.
All ligands were used in the training/testing (100:100) and the equal shape/electrostatics potential (50/50) weight was applied in the similarity scoring.
The representative PDB codes for the used X-ray crystal structures were the following: 4K6I, 1FM9, 1RDT, 1MVC, 4K4J and 3A9E for the RXRα; 4PH9, 4M11, 3QMO, 5KIR, 1PXX, 3NT1, 5JVZ and 4COX for the COX-2; 3TGE, 3HC8, 1XOZ and 1TBF for the PDE5; 5MWY, 3VHU, 2AA5, 2AA2 and 4UDA for the MR; 1B9V, 1XOG, 2QWE, 1A4Q, 2QWG, 1LTF and 6HCX for the NEU; 1L2I, 1ERR, 1XQC, 2IOK, 5TLU, 5WGD, 4XI3, 2IOG, 5KR9, 2I0G, 3ERD, 1X7R AND 3ERT for ER, and 3GBK, 6D8X, 2G0H, 2HWR, 2HFP, 2Q59, 2Q5S, 2Q8S, 2F4B, 3KMG, 2P4Y, 3BC5, 5Y2O, 3D6D, 2I4J, 2ATH, 6DGO and 1NYX for PPARγ.

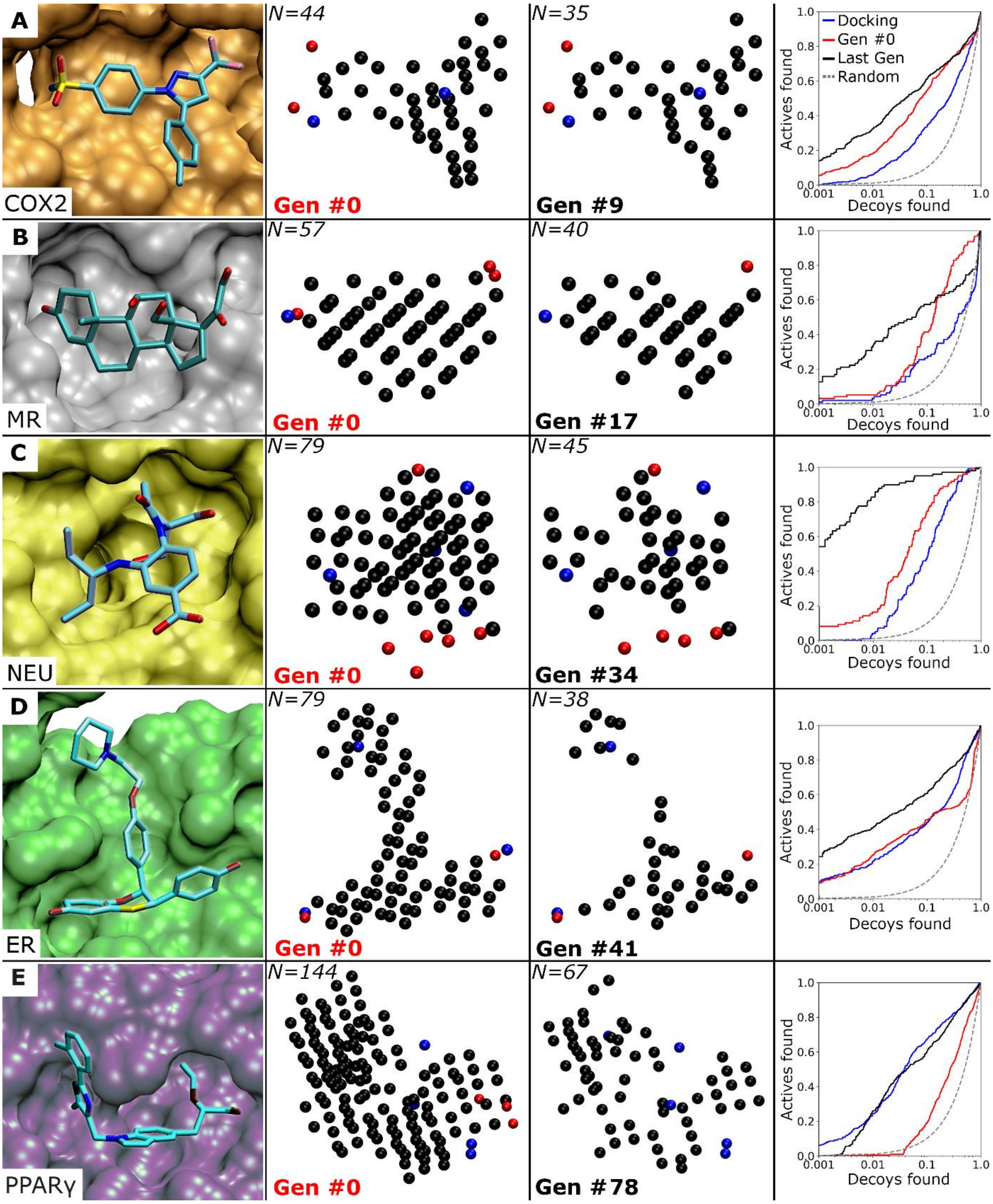

Figure S1: Negative image-based model compositions of the seven thoroughly tested DUD-E sets before and after the brute force optimization. The cavity-based input negative image-based (NIB) models (Gen #0), the brute force optimized NIB models (Gen #9-78) (100:100 in Table 1) and the semi-logarithmic receiver operating characteristic curves for A) cyclooxygenase 2 (COX-2); B) mineralocorticoid receptor (MR); C) neuraminidase (NEU); D) estrogen receptor (ER); and E) peroxisome proliferator-activated receptor gamma (PPARγ).

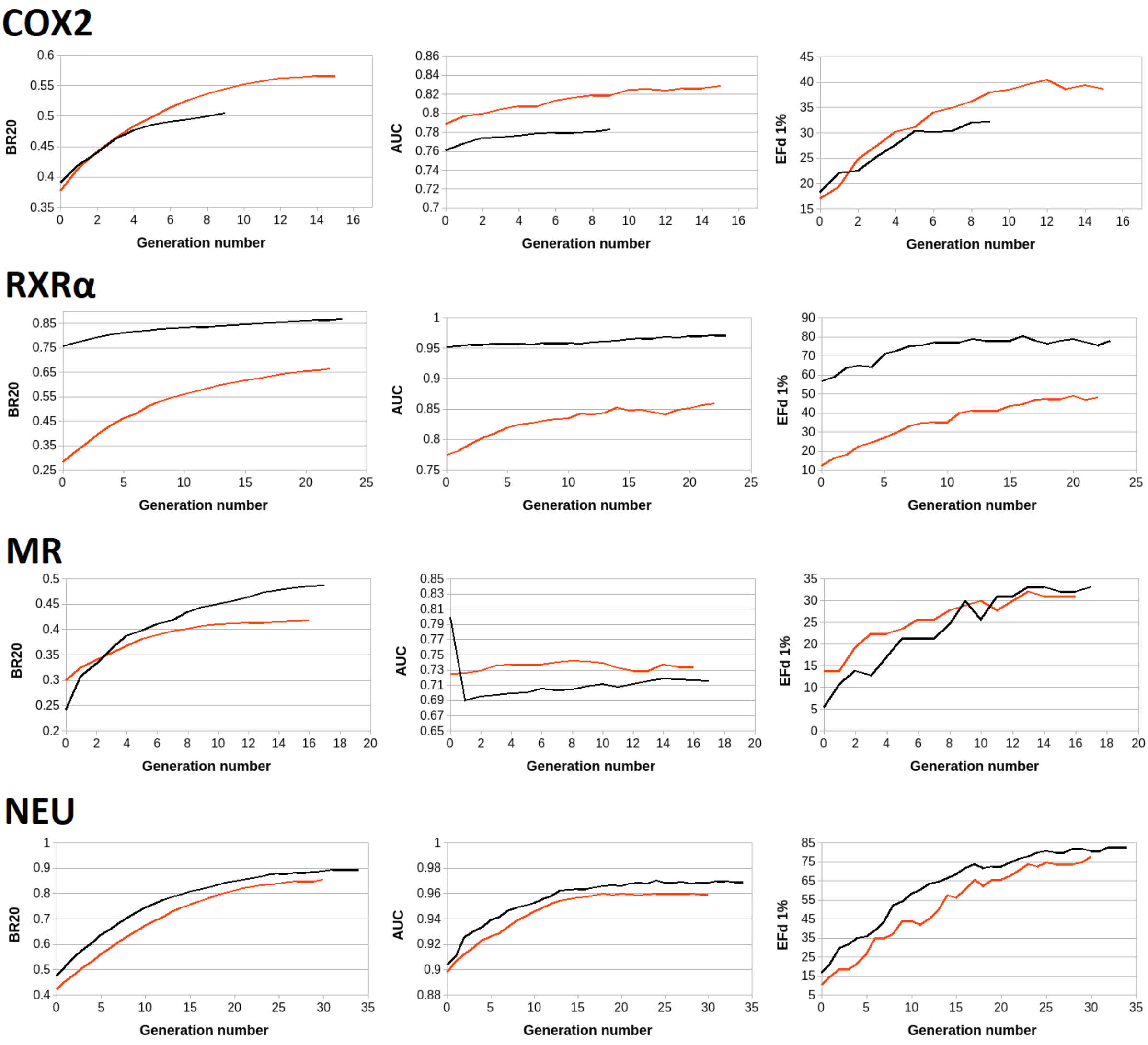

Figure S2: Evolution of enrichment metrics with four DUD-E targets during brute force optimization. The brute force negative image-based rescoring (BR-NiB) yield improvement generation-by-generation is demonstrated with BR20 (also the target metric), area under the curve (AUC) and early enrichment factor 1% (EFd 1%) for neuraminidase (NEU), cyclooxygenase 2 (COX2) and retinoid X receptor alpha (RXRα). The yield evolution is shown for equal shape/electrostatics potential (0.5 / 0.5) based optimization (black line) and for the shape only (1.0 / 0.0) based optimization (red line).

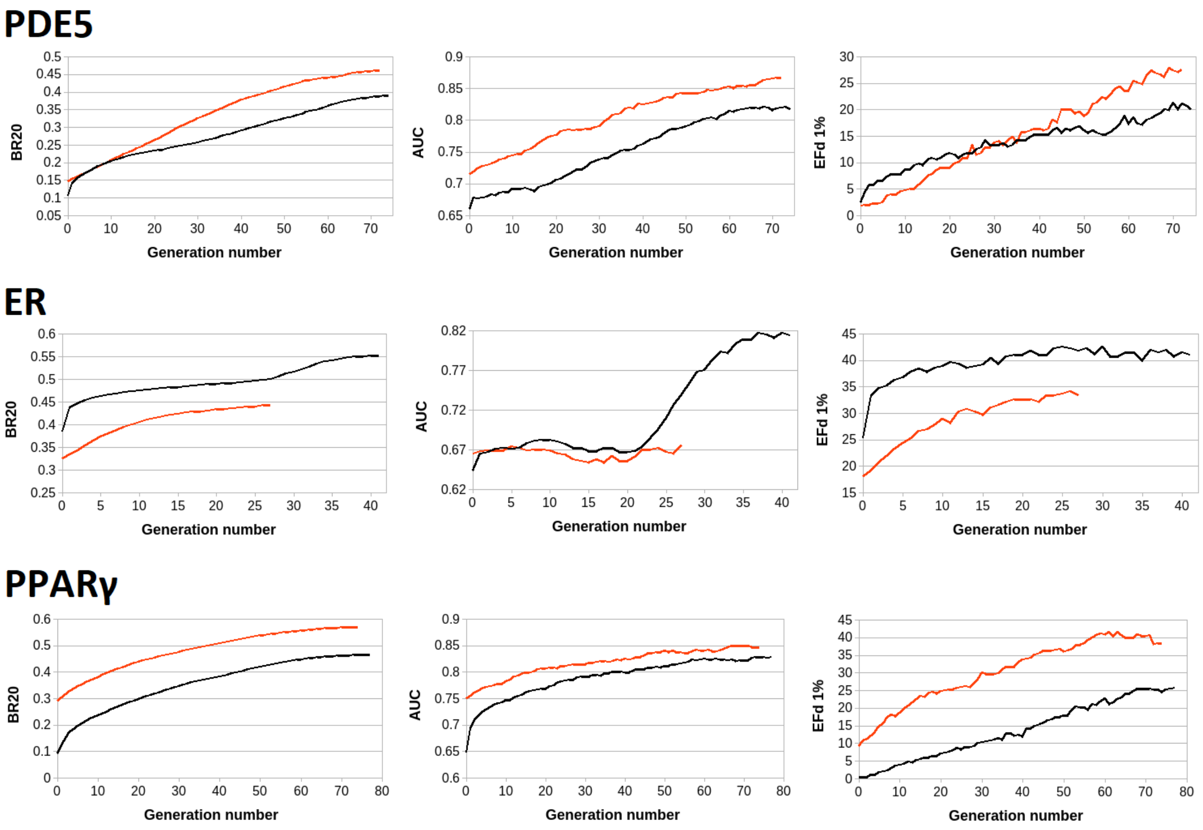

Figure S3: Evolution of enrichment metrics with three DUD-E targets during brute force optimization. The brute force negative image-based rescoring (BR-NiB) yield improvement generation-by-generation is demonstrated with BR20 (also the target metric), area under the curve (AUC) and early enrichment factor 1% (EFd 1%) for phosphodiesterase 5 (PDE5), estrogen receptor (ER), and peroxisome proliferator-activated receptor gamma (PPARγ). The yield evolution is shown for equal shape/electrostatics potential (0.5/0.5) based optimization (black line) and for the shape only (1.0 / 0.0) based optimization (red line).

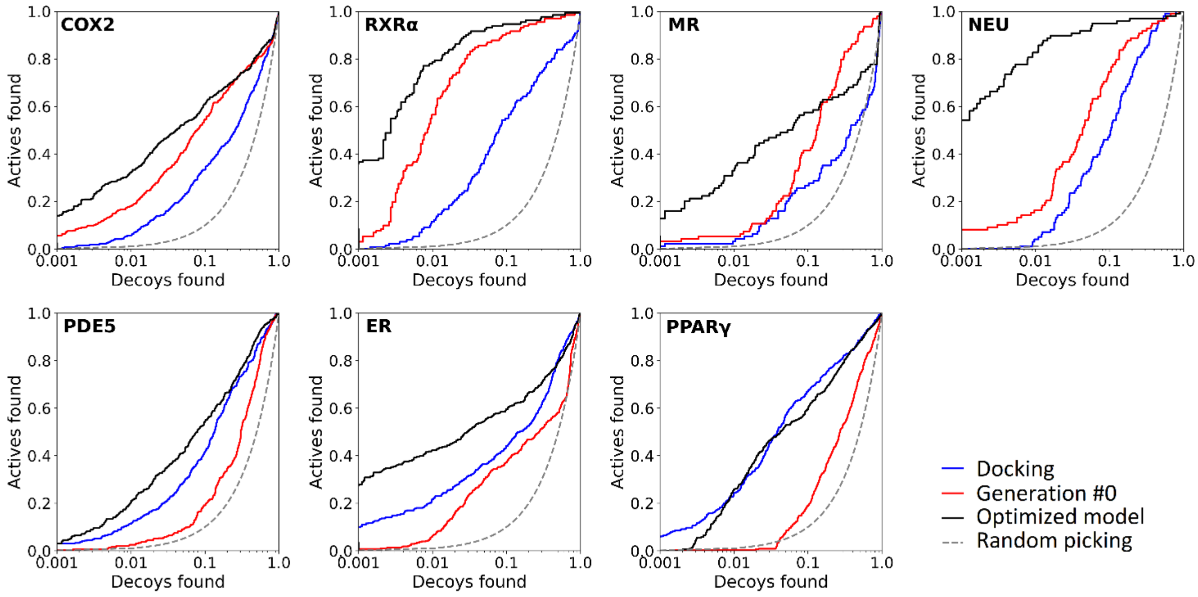

Figure S4: The semi-logarithmic receiver operating characteristic curves of the seven thoroughly tested DUD-E sets with all active ligands using equal shape/electrostatics potential in rescoring. The equivalent receiver operating characteristic (ROC) curves are shown for flexible molecular docking (PLANTS; blue line), brute force negative image-based optimization (BR-NiB; black line), and negative image-based rescoring (R-NiB) or BR-NiB generation #0 (red line) when all active ligands were applied (Table 1). Note that the BR-NiB optimized models improve the original docking enrichment consistently with every tested DUD-E (A Database of Useful (Docking) Decoys –Enhanced)[14] target (Table S1) except for peroxisome proliferator-activated receptor gamma (PPARγ).

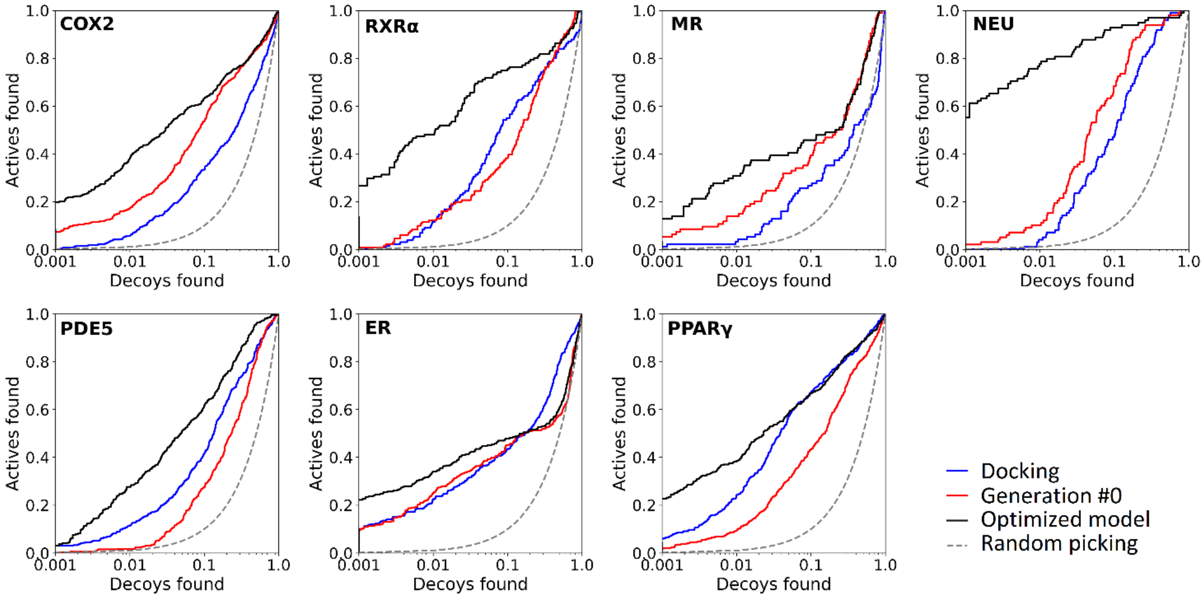

Figure S5: The semi-logarithmic receiver operating characteristic curves of the seven thoroughly tested DUD-E sets with all active ligands using only shape in rescoring. Only the x axis is logarithmic.

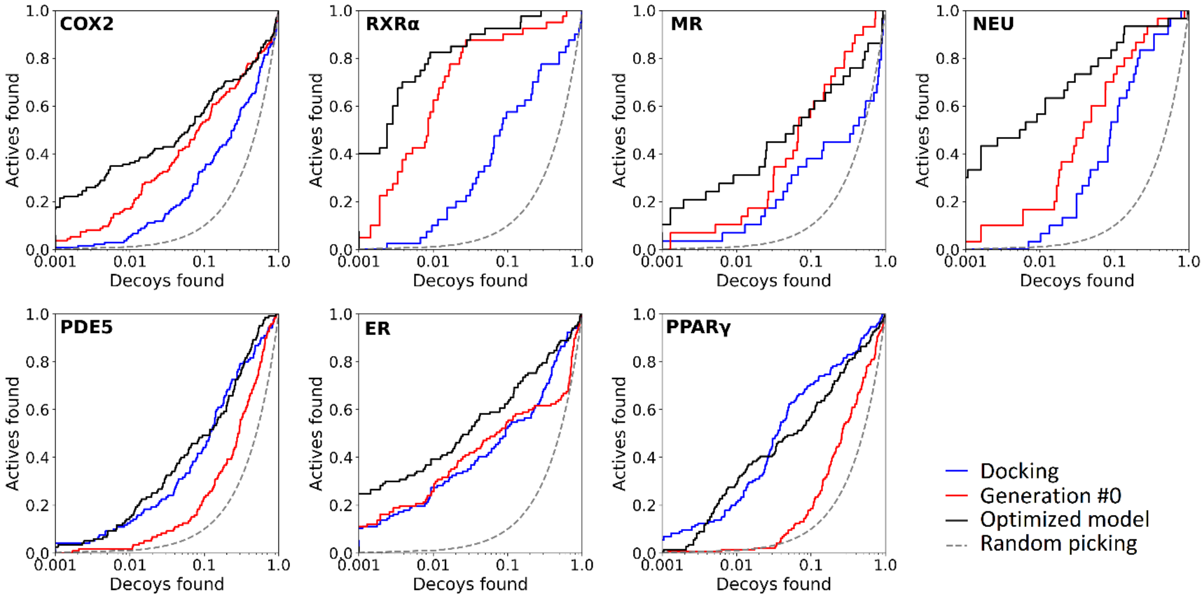

Figure S6: The semi-logarithmic receiver operating characteristic curves of the seven thoroughly tested DUD-E sets with 30 % of the active ligands using equal shape/electrostatics potential in rescoring. Only the x axis is logarithmic. The results are from the test set (70:30; Table 1) data and the training set (70:30; Table S5) data are omitted.

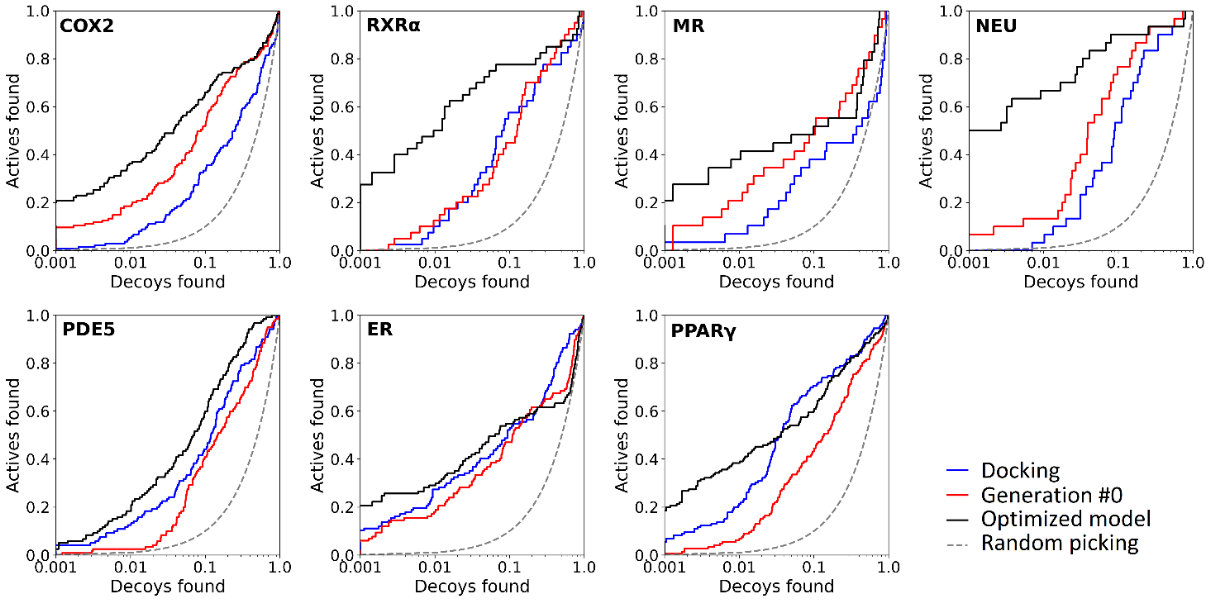

Figure S7: The semi-logarithmic receiver operating characteristic curves of the seven thoroughly tested DUD-E sets with 30 % of the active ligands using only shape in rescoring. Only the x axis is logarithmic. The results are from the test set (70:30; Table 1) data and the training set (70:30; Table S5) data are omitted.

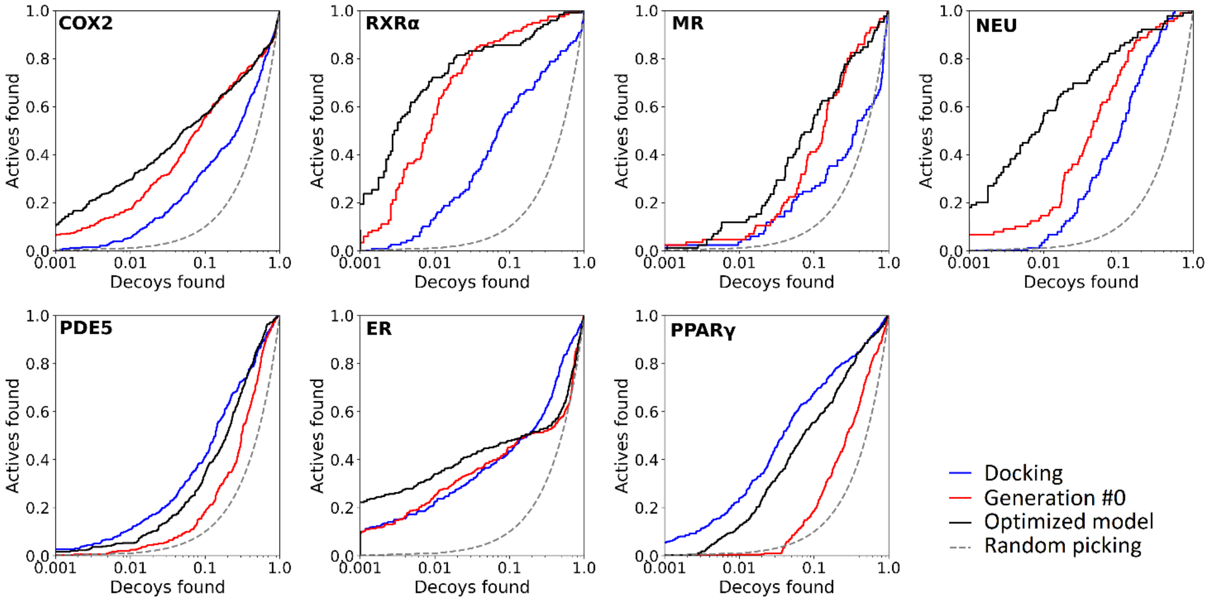

Figure S8: The semi-logarithmic receiver operating characteristic curves of the seven thoroughly tested DUD-E sets with 90 % of the active ligands using equal shape/electrostatics potential in rescoring. Only the x axis is logarithmic. The results are from the test set (10:90; Table 1) data and the training set (10:90; Table S5) data are omitted.

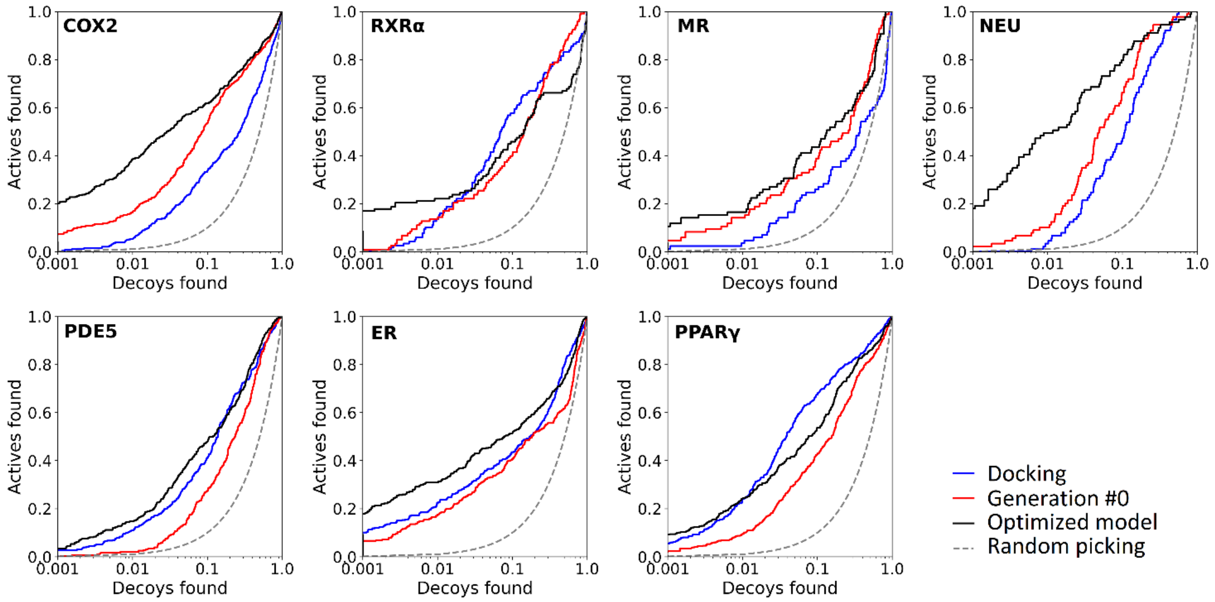

Figure S9: The semi-logarithmic receiver operating characteristic curves of the seven thoroughly tested DUD-E sets with 90 % of the active ligands using only shape in rescoring. Only the x axis is logarithmic. The results are from test set (10:<u>90</u>; Table 1) data and the training set (<u>10</u>:90; Table S5) data are omitted.

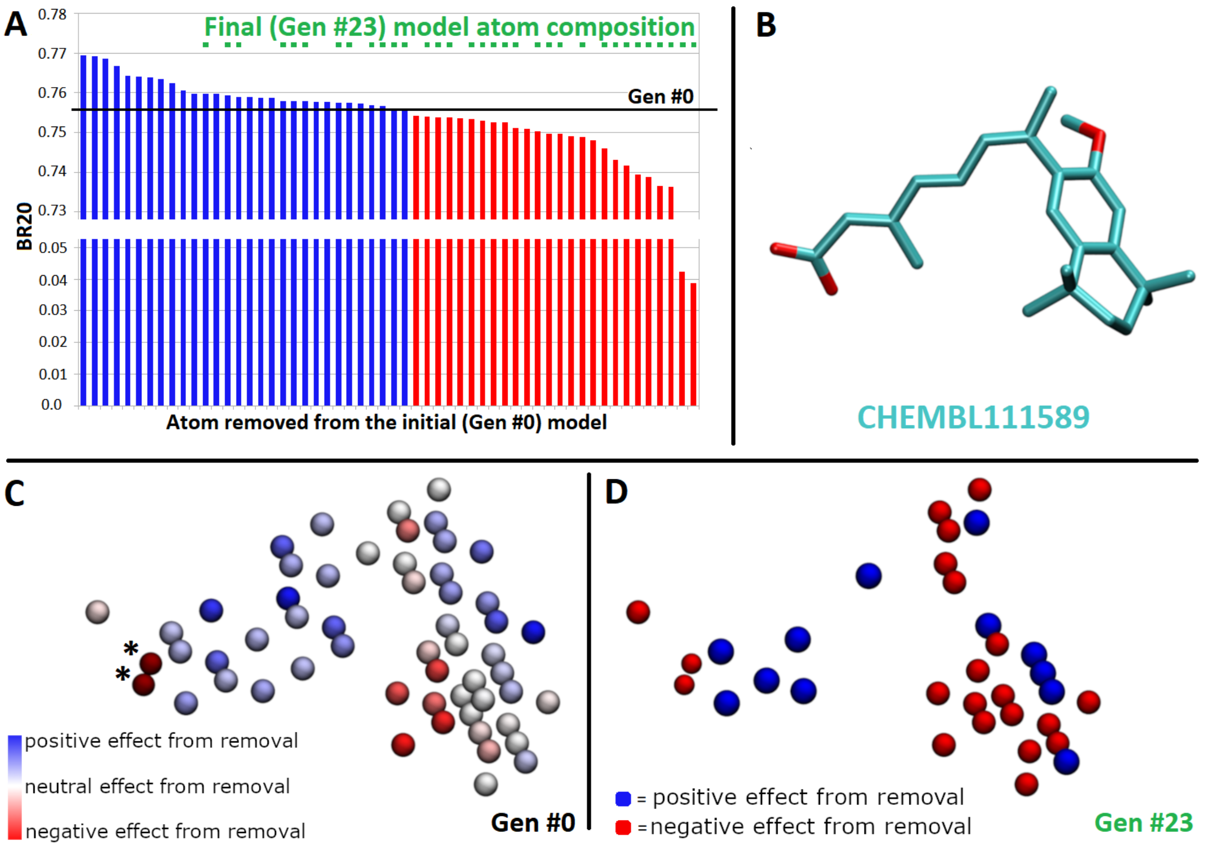

Figure S10: Generation #1 model variants – The effect of specific cavity atom removals for the retinoid X receptor alpha enrichment. A) The graph shows the BR20 values for a pool of unique NIB (negative image-based) model Gen #1 variants of retinoid X receptor alpha (N = 56; Gen #1 variants) that differ from the input model (Gen #0; black line) by one atom removal (-1 cavity atom). Green dots indicate atoms that are left in the final optimized model at Gen #23 (see panel D) after the brute force negative image-based optimization (BR-NiB; Figure 2; Videos S1 and S2). When considering the individual Gen #1 variants separately, certain atom removals improve (N = 30; blue) or lower (N = 26; red) the target enrichment BR20 value. The removal of two specific negatively charged atoms (highlighted by asterisks in panel C) worsened the yield dramatically and, in contrast, no specific removals improved enrichment in an equally large manner. Nine atoms, whose removal improved or decreased the BR20 value the most at the Gen #1 testing (red bars on the most right or blue bars on the most left), were also removed or retained, respectively, by the multi-generation optimization (Gen #23). B) The top-ranked docked pose of an active molecule CHEMBL11589 is shown as a reference. C) The input model (Gen #0) atoms are coloured according to their BR20 effect upon their removal using the RWB color scale (negative = red; neutral = white; positive = blue). D) The final optimized model (Gen #23) contains atoms, whose removal had negative (red spheres in panel A) or positive (blue spheres in panel A) effect on the enrichment at Gen #1 (no atoms considered neutral in the colouring). This indicates that the successive iterations are needed for the balanced pruning of the model and, ultimately, for acquiring the best docking rescoring results (Table 1 vs. Tables S2 and S15).

REFERENCES

1. Wang, J. L. *et al.* The novel benzopyran class of selective cyclooxygenase-2 inhibitors. Part 2: The second clinical candidate having a shorter and favorable human half-life. *Bioorganic Med. Chem. Lett.* **20**, 7159–7163 (2010).
2. Egea, P. F., Moras, D. & Biologie, L. De. Molecular Recognition of Agonist Ligands by RXRs. **16**, 987–997 (2002).
3. Bledsoe, R. K. *et al.* A ligand-mediated hydrogen bond network required for the activation of the mineralocorticoid receptor. *J. Biol. Chem.* **280**, 31283–31293 (2005).
4. Finley, J. B. *et al.* Novel aromatic inhibitors of influenza virus neuraminidase make selective interactions with conserved residues and water molecules in the active site. **4071**, 1107–1119 (1999).
5. Sung, B., Hwang, K. Y., Jeon, Y. H. & Lee, J. Il. Structure of the catalytic domain of human phosphodiesterase 5 with bound drug molecules. **425**, 98–102 (2003).
6. Card, G. L. *et al.* Structural Basis for the Activity of Drugs that Inhibit Phosphodiesterases. **12**, 2233–2247 (2004).
7. Kim, S. *et al.* Estrogen Receptor Ligands. II. Discovery of Benzoxathiins as Potent, Selective Estrogen Receptor α Modulators. *J. Med. Chem.* **47**, 2171–2175 (2004).
8. Kuhn, B. *et al.* Structure-based design of indole propionic acids as novel PPARα/γ co-agonists. *Bioorganic Med. Chem. Lett.* **16**, 4016–4020 (2006).
9. Lippa, B. *et al.* Synthesis and structure based optimization of novel Akt inhibitors. *Bioorganic Med. Chem. Lett.* **18**, 3359–3363 (2008).
10. Chien, E. Y. T. *et al.* Structure of the human dopamine D3 receptor in complex with a D2/D3 selective antagonist. *Science (80-.).* **330**, 1091–1095 (2010).
11. Rutherford, K., Le Trong, I., Stenkamp, R. E. & Parson, W. W. Crystal Structures of Human 108V and 108M Catechol O-Methyltransferase. *J. Mol. Biol.* **380**, 120–130 (2008).
12. Dvir, H. *et al.* 3D structure of Torpedo californica acetylcholinesterase complexed with huprine X at 2.1 Å resolution: Kinetic and molecular dynamic correlates. *Biochemistry* **41**, 2970–2981 (2002).
13. Roberts, W. G. *et al.* Antitumor activity and pharmacology of a selective focal adhesion kinase inhibitor, PF-562,271. *Cancer Res.* **68**, 1935–1944 (2008).
14. Mysinger, M. M., Carchia, M., Irwin, J. J. & Shoichet, B. K. Directory of useful decoys, enhanced (DUD-E): Better ligands and decoys for better benchmarking. *J. Med. Chem.* **55**, 6582–6594 (2012).
15. Hanley, J. A. & McNeil, B. J. The meaning and use of the area under a receiver operating characteristic (ROC) curve. *Radiology* **143**, 29–36 (1982).
16. Korb, O., Stützle, T. & Exner, T. E. Empirical scoring functions for advanced Protein-Ligand docking with PLANTS. *J. Chem. Inf. Model.* **49**, 84–96 (2009).
17. Hanley, A. J. & McNeil, J. B. The Meaning and Use of the Area under a Receiver Operating Characteristic (ROC) Curve. *Radiology* **143**, 29–36 (1982).
